# Excess mortality from mental, neurological and substance use disorders in the Global Burden of Disease Study 2010

**DOI:** 10.1017/S2045796014000687

**Published:** 2014-12-15

**Authors:** F. J. Charlson, A. J. Baxter, T. Dua, L. Degenhardt, H. A. Whiteford, T. Vos

**Affiliations:** 1 Queensland Centre for Mental Health Research, Wacol, Queensland, Australia; 2 University of Queensland, School of Population Health, Herston, Queensland, Australia; 3 University of Washington, Institute for Health Metrics and Evaluation, Seattle, Washington, USA; 4 World Health Organization, Department of Mental Health and Substance Abuse, Geneva; 5 University of New South Wales, National Drug and Alcohol Research Centre, New South Wales, Australia; 6 University of Melbourne, Melbourne School of Population and Global Health, Victoria, Australia

**Keywords:** Global burden of disease, mental health, mortality, neuropsychiatry

## Abstract

**Aims.:**

Mortality-associated burden of disease estimates from the Global Burden of Disease 2010 (GBD 2010) may erroneously lead to the interpretation that premature death in people with mental, neurological and substance use disorders (MNSDs) is inconsequential when evidence shows that people with MNSDs experience a significant reduction in life expectancy. We explore differences between cause-specific and excess mortality of MNSDs estimated by GBD 2010.

**Methods.:**

GBD 2010 cause-specific death estimates were produced using the International Classification of Diseases death-coding system. Excess mortality (all-cause) was estimated using natural history models. Additional mortality attributed to MNSDs as underlying causes but not captured through GBD 2010 methodology is quantified in the comparative risk assessments.

**Results.:**

In GBD 2010, MNSDs were estimated to be directly responsible for 840 000 deaths compared with more than 13 million excess deaths using natural history models.

**Conclusions.:**

Numbers of excess deaths and attributable deaths clearly demonstrate the high degree of mortality associated with these disorders. There is substantial evidence pointing to potential causal pathways for this premature mortality with evidence-based interventions available to address this mortality. The life expectancy gap between persons with MNSDs and the general population is high and should be a focus for health systems reform.

## Introduction

Findings from the Global Burden of Disease 2010 (GBD 2010) study have reinforced our understanding of the significant impact that mental, neurological and substance use disorders (MNSDs) have on population health (Murray *et al.* 2012; Whiteford *et al.* 2013). One of the key findings of GBD 2010 was the global health transition from communicable to non-communicable diseases and this is particularly rapidly in low- and middle-income countries (LMICs) (Murray *et al.* 2012). The proportion of burden attributable to non-communicable disease in LMICs has risen by more than one-third, from 36% in 1990 to 49% in 2010. In contrast, the share of non-communicable disease burden in high-income countries (HICs) has raised only 3–4% over the same time period (from 80 to 83%) (Institute of Health Metrics and Evaluation, 2013). These findings hold particular public health importance for MNSDs in LMICs for the coming decades.

GBD 2010 estimates the majority of disease burden due to MNSDs is from non-fatal health loss; only 15% of the total burden is from mortality, in terms of years of life lost (YLLs) (Institute of Health Metrics and Evaluation, 2013). This may erroneously lead to the interpretation that premature death in people with mental and neurological disorders is inconsequential, whereas evidence shows that people with MNSDs experience a significant reduction in life expectancy (Chang *et al.* 2011; Wahlbeck *et al.* 2011; Crump *et al.* 2013; Lawrence *et al.* 2013). In Australia and the UK males with a mental disorder die, on average, 15 years earlier than the general population and females die on average 12 years earlier (Crump *et al.* 2013; Lawrence *et al.* 2013). It is estimated that about 80% of premature deaths in people with MNSDs are due to physical illnesses, particularly cardiovascular disease, including stroke and cancer (Crump *et al.* 2013; Lawrence *et al.* 2013). Dementia is an independent risk factor for premature death with increased risk found in those patients with physical impairment and inactivity, and medical comorbidities (Park *et al.* 2014). Excess mortality in people with epilepsy is reported to be two- to threefold higher compared with the general population; with an increased risk of up to sixfold higher in LMICs (Diop *et al.* 2005). A significant proportion of these deaths are preventable, resulting from falls, drowning, burns and status epilepticus (Diop *et al.* 2005; Jette & Trevathan, 2014). A recent review has shown the highest standardised mortality ratio (SMR) among mental and substance use disorders was 14.7 for opioid use disorders (Chesney *et al.* 2014). In HICs the life expectancy gap is widening with the general population now enjoying a longer life while the lifespan for those with a mental disorder has remained static (Lawrence *et al.* 2013).

Mortality associated with a disease can be quantified using two different, yet complementary, methods which are employed as part of GBD analyses. First, cause-specific mortality draws upon vital registration systems and verbal autopsy studies which identify deaths attributed to a single underlying cause using the International Classification of Diseases (ICD) death-coding system. Second, GBD creates natural history models for each disease, including its distribution across age and sex. This involves estimation of a range of epidemiological parameters, including excess mortality – that is, the all-cause mortality rate in a population with the disorder compared with the all-cause mortality rates in a population without the disorder. By definition, estimates of excess deaths include cause-specific deaths.

Although often arbitrary, the ICD conventions are a necessary attempt to deal with the multi-causal nature of mortality and avoid ‘double-counting’ of deaths. However, despite the systems clear strengths, cause-specific mortality estimated via the ICD obscures the contribution of other underlying causes of death; for example, suicide as a direct result of major depressive disorder coded as injury, and will likely underestimate the true number of deaths attributable to a particular disease. On the other hand, estimation of excess mortality using natural history models will often comprise deaths from both causal and non-causal origins and will likely overestimate the true number of deaths attributable to a particular disorder. The challenge is to parse out causal contributions to mortality (beyond those already identified as cause-specific) from the effects of confounders.

Quantification of the contributions of multiple causal factors to excess mortality associated with a particular disease is challenging and requires approaches such as the comparative risk assessment (CRA), which is now an integral part of the GBD studies. The fundamental approach for the GBD CRA is to calculate the proportion of deaths or disease burden caused by specific risk factors – e.g., lung cancer caused by tobacco smoking – while holding all other independent factors unchanged. A key concept when attempting to quantify causal relationships is that of ‘counterfactual burden’ which compares the burden associated with an outcome with the amount that would be expected in a hypothetical situation of ‘ideal’ risk factor exposure (e.g., zero prevalence). This approach provides a consistent method for estimating the changes in population health as a function of decreasing or increasing the level of exposure to risk factors (Lim *et al.* 2012). Importantly, the flexibility within counterfactual analysis allows the sum of death counts attributed to different risk factors for a particular cause to sum to more than 100% which is not permissible by ICD registry data.

In this paper, we explore the cause-specific and excess mortality of individual MNSDs estimated by GBD 2010. We also present the additional attributable burden that can be ascribed to disorders using GBD results for CRA's assessing MNSDs as risk factors for other health outcomes. Disorders included in the analyses are grouped by: mental disorders (schizophrenia, major depression (MDD), anxiety disorders, bipolar disorder, childhood behavioural disorders (attention-deficit/hyperactivity disorder (ADHD) and conduct disorder (CD)), autistic disorder and intellectual disability); substance use disorders (alcohol, opioid, cocaine and amphetamine use disorders); and neurological disorders (dementia, epilepsy and migraine).

## Methods

### YLLs and cause of death

The GBD 2010 methodology uses a time-based metric, YLLs, to quantify the fatal burden by underlying cause (Lozano *et al.* 2012). YLLs are computed by multiplying the number of deaths attributable to a particular disease at each age by a standard life expectancy at that age. The standard life expectancy represents the normative goal for survival and for 2010 was computed based on the lowest recorded death rates in any age group in countries with a population greater than 5 million (Salomon *et al.* 2012).

Cause-specific death estimates in GBD 2010 were produced from available cause of death data for 187 countries from 1980 to 2010. Data sources included vital registration, verbal autopsy, mortality surveillance, censuses, surveys, hospitals, police records and mortuaries (Lozano *et al.* 2012). Cause of death ensemble modelling (CODEm) was used for all MNSDs. In summary, CODEm uses four families of statistical models testing a large set of different models using different permutations of covariates. Model ensembles were developed from these component models and model performance was assessed with rigorous out-of-sample testing of prediction error and the validity of 95% uncertainty intervals (UIs). Details relating to CODEm and the method for how these models were used in calculating YLLs are described in detail elsewhere (Lozano *et al.* 2012).

YLLs for GBD 2010 were computed from cause-specific mortality estimates for seven of the 15 MNSDs: schizophrenia; opioid, amphetamine, cocaine and alcohol use disorders; dementia and epilepsy (Lozano *et al.* 2012). As the ICD does not permit the other mental and neurological disorders to be recorded as the ‘primary’ cause of death, YLLs were unable to be calculated for the remaining eight disorder groups (World Health Organization, 1993; Lim *et al.* 2012).

### Excess mortality from a natural history model

Drawing on a series of systematic reviews, we collated comprehensive sets of epidemiologic data for each disorder. Data were pooled, adjusting for between-study variance, and then an internally consistent epidemiologic model derived using the relationship described in the generic disease model (see the appendix) (Vos *et al.* 2012). To do this we used DisMod-MR, a Bayesian meta-regression tool which estimates a natural history of disease model producing age-, sex- and region-specific estimates for prevalence, incidence, remission and excess mortality (Vos *et al.* 2012). Where data were scarce, DisMod-MR was able to impute information with associated uncertainty ranges based on epidemiologically and geographically similar populations. Excess mortality estimates based on this natural history model are reported for MNSDs in terms of global deaths for 2010. Further details of the GBD 2010 methods for developing a natural history model of disease using DisMod-MR have been described in detail elsewhere (Vos *et al.* 2012).

### Counter-factual burden and CRA

Prince *et al.* (2007) have summarised the evidence where a causal relationship between mental and substance use disorders and other health outcomes have been proposed. In GBD 2010, a series of reviews were conducted to assess the strength of evidence for MNSDs as independent risk factors for other health outcomes (Degenhardt *et al.* 2009*a*; Rehm *et al.* 2010*a*; Charlson *et al.* 2011; Degenhardt & Hall, 2012). Risk factor studies were identified through systematic searches of published and unpublished data with information on effect sizes and study characteristics extracted and collated (Charlson *et al.* 2013; Degenhardt *et al.* 2013; Ferrari *et al.* 2014). A meta-synthesis was used to calculate a relative risk (RR) for MNSDs (the exposure) as a risk factor for other health outcomes. The RR was then applied to prevalence distributions of the specific exposures by sex and age-group for each geographic region to derive population attributable fractions (PAFs). More detail on the calculation of PAFs in GBD 2010 is provided by Lim *et al.* (2012). In some cases, for example suicide, ceiling values were calculated and applied for joint PAFs to ensure the sum of proportional contribution for all risk factors did not exceed 100% (Ferrari *et al.* 2014). The additional burden (YLLs and YLDs) attributable to MNSDs is the product of the PAFs and the burden for the health outcome as estimated in GBD 2010.

Here we compare the number of cause-specific deaths reported (YLLs) for MNSDs, calculated as part of the GBD 2010 study with the number of all-cause deaths derived from natural history models. We explore differences in these estimates across the age-span for each disorder. Additional YLLs attributed to disorders as underlying causes and quantified in the CRA are reported.

## Results

### YLLs and causal mortality

Globally, the seven disorders (dementia, epilepsy, schizophrenia, alcohol use disorders, opioid use disorders, cocaine use disorders and amphetamine use disorders) for which YLLs were estimated were directly responsible for 1.3 million deaths in 2010, equating to about 12 million YLLs (see Fig. 2 in the appendix). Epilepsy and then dementia contributed the greatest proportion of YLLs within this group.

**Fig. 1.**
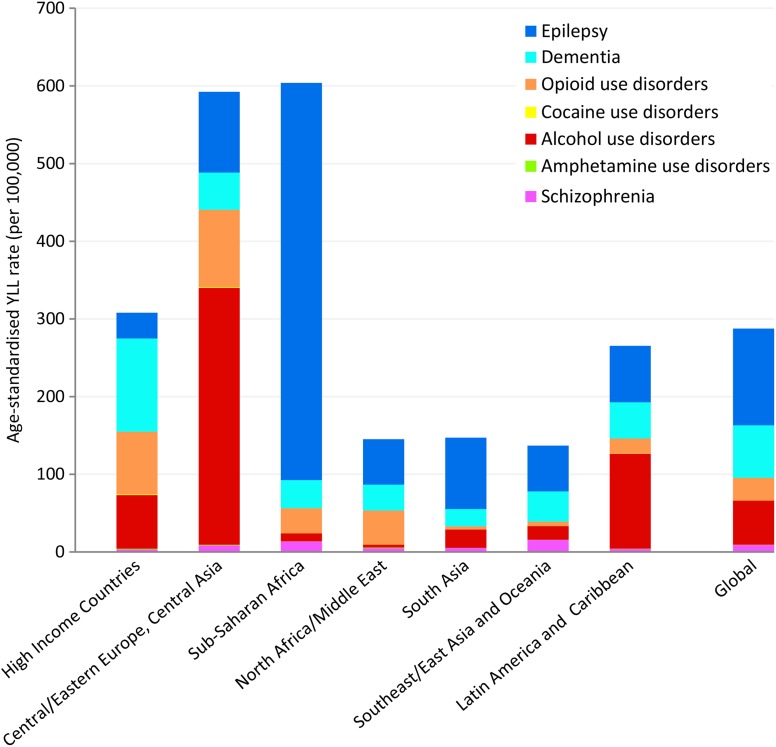
Age-standardised YLL rates (per 100 000 population) for MNSDs by GBD super-region and disorder, 2010.

Age-standardised YLL rates vary considerably across the seven geographical super-regions primarily due to differences in patterns of alcohol and drug use, and mental and neurological disorder prevalence. There are several regions with substantial deviations from global YLL average rates (Fig. 1). (Details of which countries are in each sup-region can be found on the IHME website (Institute for Health Metrics and Evaluation, 2014).)

In 2010, YLL rates were highest in the sub-Saharan Africa region (604 YLLs per 100 000 population) and the region comprising Central/Eastern Europe plus Central Asia (593 YLLs per 100 000); the causes for which vary considerably (Fig. 1). In sub-Saharan Africa the YLL burden was driven by epilepsy which was fourfold higher than the global average and approximately 85% of all YLLs attributed to MNSDs in sub-Saharan Africa. Although the substance use disorders YLL rates appear unremarkable for this region, their YLL burden has increased 3.0% from 1990 to 2010, almost double the average global increase and the highest of all regions (Degenhardt *et al.* 2013). In contrast to sub-Saharan Africa, the high fatal burden in Central/Eastern Europe and Central Asia was largely caused by deaths attributed to alcohol use. High mortality due to illicit substance use disorders also contributed to the YLL rate in Central/Eastern Europe and Central Asia with all substance use disorders together explaining 73% of YLLs in the region. Countries within East Asia and the Pacific exhibit very low YLL rates across all MNSDs with little change observed between 1990 and 2010.

**Fig. 2.**
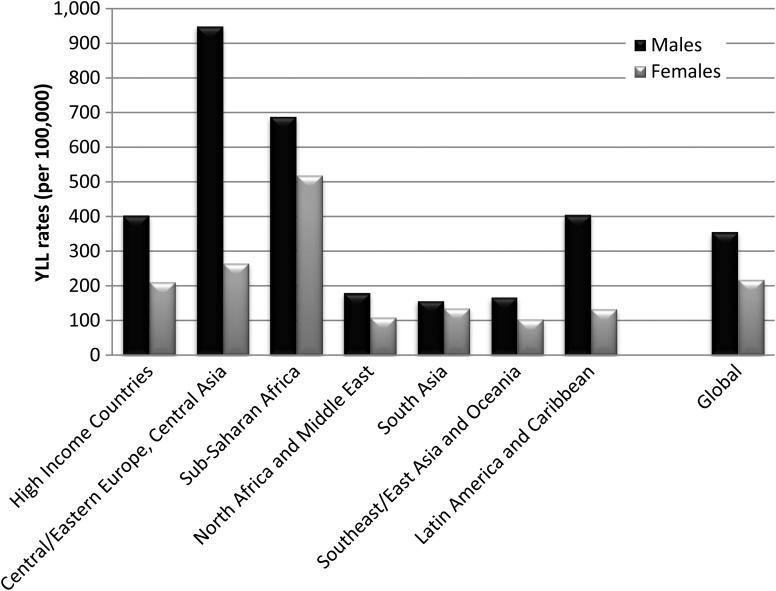
Age-standardised YLL rates (per 100 000) for MNSDs by GBD super-region and sex, 2010.

Globally, neurological disorders accounted for 58% of the all MNSD YLLs in males and 81% of YLLs in females. Substance use disorders explained 39% of YLLs in males and 16% of those in females. The contribution of schizophrenia to total MNS disorder YLLs was similar for both genders (3% each). Differences in YLL patterns between the genders were influenced in part by the differing contribution to YLLs by substance use disorders compared with neurological disorders across regions (Fig. 2).

### Excess mortality from a natural history model

The only mental disorder for which cause-specific deaths and YLLs were estimated in GBD was schizophrenia; however, several mental disorders, such as major depression and bipolar disorder, exhibit significant and documented excess-mortality (Roshanaei-Moghaddam & Katon, 2009; Baxter *et al.* 2011*b*) (Table 1). There were four disorders for which sufficient evidence of excess all-cause mortality could not be found in the literature (anxiety, childhood behavioural disorders, cannabis dependence and migraine) and therefore excess mortality was not included in the natural history of disease for these disorders.

**Table 1.** Presence of cause-specific mortality and excess mortality attributed to DCP3 MNSDs in GBD 2010

Disorders	Cause-specific mortality attributed to disorders in GBD 2010	Excess mortality attributed to disorders in GBD 2010
Mental disorders		
Major depressive disorder	No	Yes
Anxiety disorders	No	No
Schizophrenia	Yes	Yes
Bipolar disorders	No	Yes
Childhood behavioural disorders (ADHD & CD)	No	No
Autistic disorder	No	Yes
Intellectual disability	No	Yes
Substance use disorders		
Alcohol use disorders	Yes	Yes
Opioid dependence disorder	Yes	Yes
Cannabis dependence disorder	No	No
Amphetamine dependence disorder	Yes	Yes
Cocaine dependence use disorder	Yes	Yes
Neurological disorders		
Epilepsy	Yes	Yes
Migraine	No	No
Dementia	Yes	Yes

ADHD, attention-deficit/hyperactivity disorder; CD, conduct disorder.

### Mental disorders

**Fig. 3.**
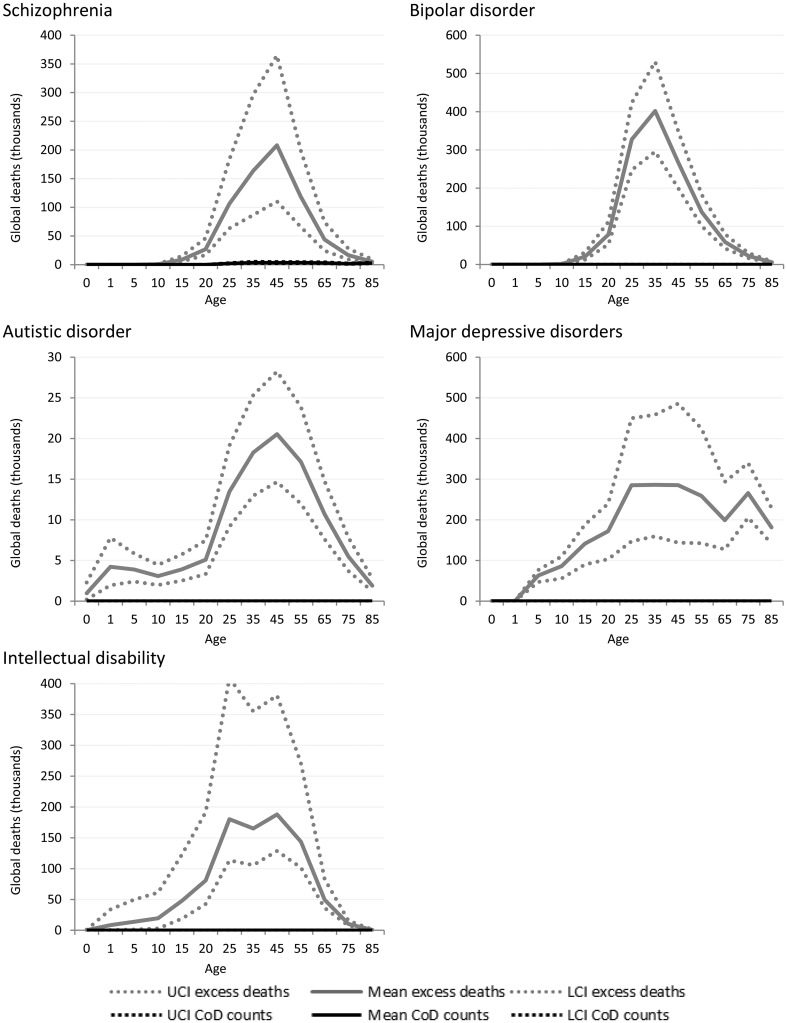
Numbers of cause-specific and excess deaths attributed to mental disorders in 2010, by age with upper and lower 95% CI. *Note: ADHD, CD and anxiety not shown as there were no cause-specific or excess mortality estimated (Table 1).

Figure 3 shows the estimated number of cause-specific and excess deaths for each of the five mental disorders with estimated excess mortality by age and with uncertainty bounds. While cause-specific deaths were attributed to only one mental disorder (schizophrenia), excess mortality was present in natural history models for five: schizophrenia, bipolar disorder, MDD, autistic disorder and intellectual disability.

Although schizophrenia is one of the few mental disorders with cause-specific deaths permissible by ICD, the numbers of cause-specific deaths globally (approximately 20 000) are noticeably lower compared with all-cause deaths (approximately 700 000) ascribed by the disorder's natural history. Around 1.3 million excess deaths are estimated in the natural history model of bipolar disorder but there are no cause-specific deaths attributed to the disorder. The natural history of the disease suggests, however, that bipolar disorder is associated with a higher number of excess deaths globally than schizophrenia. No deaths were coded to depressive disorders in GBD 2010. Natural history models of MDD suggest there were more than 2.2 million excess deaths in persons with MDD, with a particularly high rate of death in older persons that is not observed in schizophrenia or bipolar disorder.

Intellectual disability was modelled as an ‘envelope disorder’ for GBD 2010, meaning that the intellectual disability ascribed to all underlying causes including meningitis, Down's syndrome, and chromosomal defects, were captured under a single disorder category. After modelling, the contributions of each specific underlying cause were separated out and a ‘rest’ category of idiopathic intellectual disability was created. There were no deaths causally attributed to intellectual disability; however, excess deaths in people with idiopathic intellectual disability were estimated to be substantial at over 900 000 deaths globally in 2010.

At this time there is insufficient information available to determine whether premature mortality is significantly raised across the spectrum of anxiety disorders (Baxter *et al.* 2014) and in childhood behavioural disorders (Erskine *et al.* 2013). In GBD 2010 there were no YLLs or excess mortality associated with the natural history of disease applied to anxiety disorders or childhood behavioural disorders.

### Substance use disorders

**Fig. 4.**
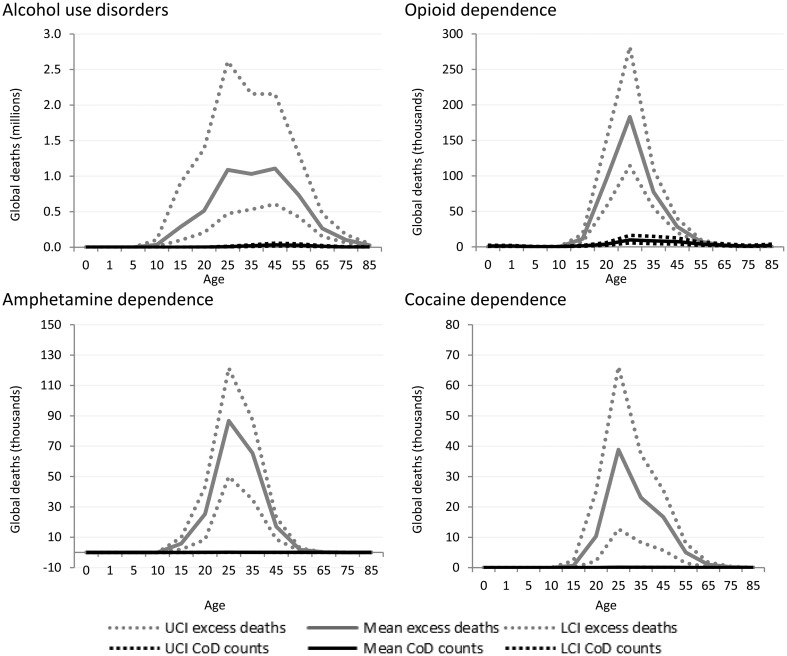
Numbers of cause-specific and excess deaths attributed to substance use disorders in 2010, by age and with upper and lower 95% CI. *Note: Cannabis not shown as there was no cause-specific or excess mortality.

GBD estimates indicate more than 110 000 deaths were causally attributed to alcohol use disorders worldwide in 2010, but indicative of the true impact of alcohol dependence as an underlying cause of death in many is that over 5 million excess deaths were estimated in the same year. Over 700 000 excess deaths occurred in dependent illicit drug users in 2010 compared with only 44 000 deaths which were coded as the cause of death. The majority of these deaths can be ascribed to opioid dependence (43 000) (Fig. 4).

### Neurological disorders

**Fig. 5.**
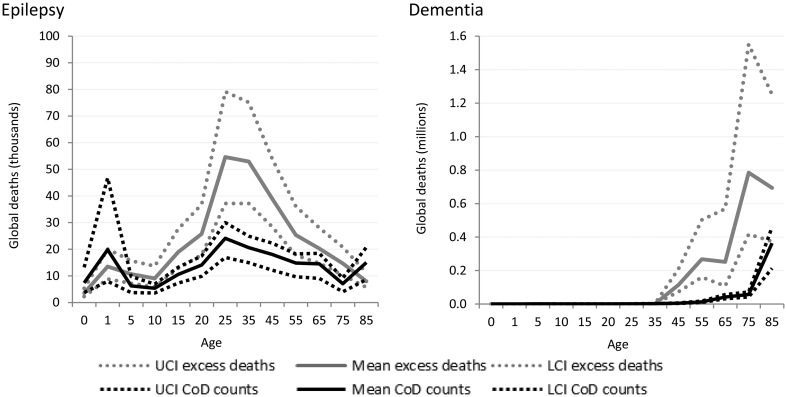
Numbers of cause-specific and excess deaths attributed to neurological disorders in 2010, by age and with upper and lower 95% CI. *Note: Migraine not shown as there was no cause-specific or excess mortality.

Cause-specific death estimates are more substantial for neurological disorders (Fig. 5) resulting in a less dramatic gap between cause-specific and excess deaths. This is likely indicative of neurological disorders being recognised more readily as the primary cause of death.

Similar to intellectual disability, epilepsy was modelled as an envelope disorder in GBD 2010 with idiopathic epilepsy and epilepsy secondary to a range of causes, including meningitis, neonatal tetanus, iodine deficiency and a variety of birth complications, being modelled as one disorder. Cause of death modelling estimated nearly 180 000 deaths due to epilepsy in 2010 while natural history models show us about 300 000 excess deaths in fact took place. The proportion of deaths attributable to different causes differ by region and GBD 2010 showed sub-Saharan African populations had the highest death rate due to epilepsy. Around 2.1 million excess deaths worldwide were estimated from dementia for 2010, yet less than 500 000 were attributed to dementia as the primary cause of death.

Table 2 shows that the cause-specific deaths and excess deaths directly coded to MNSDs are relatively similar up to 4 years of age but then rise sharply: in children aged 5–9 years there were 7420 cause-specific deaths compared with more than 91 000 excess deaths in the same age group. Alcohol use disorders explained the highest number of excess deaths (5.2 million): 38% of all excess deaths due to mental and neurological disorders in 2010. Considered together, the mental disorders for which no cause-specific deaths were attributed (bipolar disorder, major depression, autism and intellectual disability) explained more than 4.5 million deaths, equating to one third of all excess deaths in 2010.

**Table 2.** Number of cause-specific and excess deaths, by age for 2010

	0–1 years	1–4 years	5–9 years	10–14 years	15–19 years	20–24 years	25–34 years	35–44 years	45–54 years	55–64 years	65–74 years	75+ years	Total
Cause-specific deaths													
Alzheimer's disease and other dementias	–	–	869	605	578	642	1259	2302	4575	12 559	41 622	420 710	485 721
Epilepsy	7388	19 819	6255	5351	10 562	14 101	24 107	20 605	18 038	14 826	14 522	22 054	177 627
Schizophrenia	–	–	–	–	–	–	2003	3610	3429	3440	3035	4246	19 763
Alcohol use disorders	–	–	–	–	464	1311	7937	20 044	33 613	27 446	13 295	7024	111 134
Opioid use disorders	1231	1217	288	260	1350	3745	9736	8446	7432	3846	2319	3171	43 040
Cocaine use disorders	13	12	3	3	16	47	120	107	96	53	33	44	549
Amphetamine use disorders	13	11	3	3	14	40	102	88	75	44	30	41	465
Total	8644	21 059	7420	6222	12 984	19 886	45 263	55 201	67 258	62 214	74 856	457 291	838 299
Excess deaths													
Alzheimer's disease and other dementias	–	–	–	–	–	–	–	1160	114 334	267 613	251 719	1 478 957	2 113 783
Epilepsy	3513	13 486	10 680	9050	18 957	25 784	54 590	52 928	38 961	25 330	20 276	22 647	296 201
Schizophrenia	–	–	–	816	8758	26 990	106 121	163 634	208 056	118 828	43 846	21 945	698 993
Alcohol use disorders	–	–	–	33 178	285 509	512 200	1 088 632	1 030 419	1 107 039	734 711	265 061	130 643	5 187 391
Opioid use disorders	–	–	–	–	11 268	94 748	183 102	77 352	28 489	7350	1498	319	404 125
Cocaine use disorders	–	–	–	–	638	10 334	38 838	23 083	16 682	5023	984	237	95 818
Amphetamine use disorders	–	–	–	–	5856	25 306	86 702	65 420	17 058	1765	101	11	202 219
Other mental disorders with excess deaths*	1136	12 821	80 771	110 192	214 234	336 637	806 074	871 155	759 926	556 262	317 845	493 689	4 560 741
Total	4649	26 307	91 451	153 236	545 220	1 032 000	2 364 057	2 285 150	2 290 545	1 716 881	901 329	2 148 447	13 559 272

Note: larger than expected numbers in the 75+ age group may be an artefact of age groupings.

*Disorders with all-cause excess mortality but no cause-specific mortality (bipolar, autistic disorder, major depression and intellectual disability).

### Counter-factual burden and CRA

The reviews conducted as part of GBD 2010 collectively yielded sufficient evidence for several CRAs (see Table 3). Neurological disorders were not assessed as risk factors in GBD 2010.

**Table 3.** MNSDs included as risk factors in GBD 2010 CRAs with attributable YLLs for health outcomes in 2010

Risk	Outcome	YLLs in thousands (95% CI)
Alcohol use	Alcohol use disorders, tuberculosis, lower respiratory infections, multiple cancers, cardiovascular and circulatory diseases, cirrhosis of the liver, pancreatitis, epilepsy, diabetes mellitus, injuries and interpersonal violence*	78 706 (70 946–86 778)
Injecting drug use	HIV/AIDS, Hepatitis B and C, liver cancer and cirrhosis of the liver secondary to hepatitis	7233 (5646–9693)
Mental and substance use disorders	Suicide	22 504 (14 804–29 783)
Major depression	Ischaemic heart disease	3573 (1791–5412)
Cannabis dependence	Schizophrenia	Nil

*Source: Table 1. From Lim, Vos, Flaxman *et al.* A comparative risk assessment of burden of disease and injury attributable to 67 risk factors and risk factor clusters in 21 regions, 1990–2010: a systematic analysis for the Global Burden of Disease Study 2010. The Lancet no. 380 (9859):2224–2260.

For mental disorders, a number of associations were investigated but data limitations meant that only suicide and IHD were able to be included as outcomes for mental disorders (suicide) and MDD (IHD) (Baxter *et al.* 2011*a*; Charlson *et al.* 2011; Ferrari *et al.* 2014). Collectively, mental and substance use disorders are estimated to be responsible for about 22 million YLLs due to death by suicide (Ferrari *et al.* 2014). The CRA of major depression as a risk factor for ischaemic heart disease estimated an attributable to burden of about 3.5 million YLLs (Charlson *et al.* 2013).

Injecting drug use was considered as a risk factor for a number of outcomes, including blood borne viruses and liver disease, and collectively accounted for over 7 million YLLs in attributable burden. Interestingly, and despite common preconceptions, GBD results did not show any mortality-related burden from schizophrenia that could be attributed to cannabis dependence. Alcohol use was the biggest contributor with nearly 80 million attributable YLLs estimated across a number of health outcomes.

**Fig. 6.**
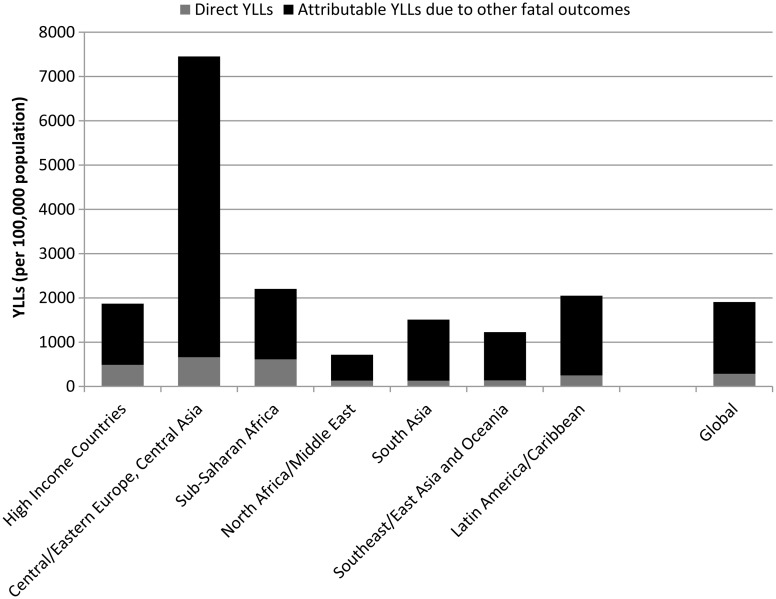
YLL rates (per 100 000 population) for deaths directly associated with MNSDs and indirect deaths for MNSDs as risk factors for other health outcomes, 2010. Note: Indirect deaths include deaths attributable to alcohol and drug use from CRA study; suicide deaths attributable to mental and substance use disorders; and ischaemic heart disease deaths attributable to major depression (see Table above for specific fatal outcomes)

Figure 6 shows the considerable additional burden when MNSDs are considered as underlying contributors to other health outcomes. Given the large estimate of mortality-related burden attributed to alcohol dependence, it is expected that, when aggregating YLLs, the regions with the largest attributable burden will be those which have highest rates of alcohol dependence, i.e., Eastern Europe and Central Asia. Sub-Saharan Africa experiences large communicable disease YLLs attributable to alcohol dependence as a result of the continuing high prevalence of communicable disease in relation to other regions.

By incorporating the additional YLLs estimated using CRAs into the overall contribution of mental, substance use and neurological disorders to all cause YLLs (Table 4) we can see a dramatically different picture to that painted in Appendix 1 where YLL contributions appeared negligible in many cases.

**Table 4.** Revised MNS disorder YLLs (per 100 000 population) as a % of all cause YLLs after the inclusion of CRA burden estimates

Region	Direct YLLs	Attributable YLLs due to other fatal outcomes	Total	Percentage of all cause YLLs (%)
High income countries	487	1384	1871	20
Central/Eastern Europe, Central Asia	661	6791	7452	34
Sub-Saharan Africa	613	1591	2204	5
North Africa/Middle East	132	584	716	4
South Asia	130	1380	1510	5
Southeast/East Asia and Oceania	137	1090	1227	7
Latin America/Caribbean	248	1803	2051	10
Global	286	1622	1908	8

Contributions across regions vary in accordance with the epidemiological profile of disorders within each region, not only of mental, substance use and neurological disorders but also the health outcomes assessed in CRAs. For example, the relatively large contribution in HICs and Central/Eastern Europe and Central Asia likely to be reflective not only of high prevalence of substance use, but also of cardiovascular disease (CVD) which was assessed as an outcome of major depression and alcohol use. In contrast, the relatively lower contribution in sub-Saharan Africa is likely reflective of comparatively lower rates of both substance use disorders (risk factors) and chronic diseases such as CVD (health outcomes). If neurological disorders were assessed as risk factors for other health outcomes using CRAs this picture may have looked different for sub-Saharan Africa where YLLs attributable to neurological disorders is higher than in other regions in the world.

## Discussion

A relatively small YLL burden was attributable to MNSDs in GBD 2010; however, numbers of excess deaths derived from natural history of disease models clearly demonstrate the high degree of mortality associated with these disorders. Quantifying the independent contributions of mental and substance use disorders to poor health outcomes through methods such as the CRA is restricted by data availability and methodological challenges such as establishing causal relationships (Baxter *et al.* 2011*a*); nevertheless, there is a growing body of literature which can help us develop hypotheses around these contributions by observing the risks associated with excess death in individuals with mental and substance use disorders.

The relationship between mental disorders and suicide has long been recognised (Li *et al.* 2011). Mental disorders have also been linked to higher rates of death due to coronary heart disease, stroke, type II diabetes, respiratory diseases and some cancers (Hoyer *et al.* 2000; Crump *et al.* 2013). The relationship between mental disorders and physical disease, leading to premature death, is complex. People with mental disorders have an increased risk of death in several ways, for example people with MDD are more likely to develop CVD (Charlson *et al.* 2011). Psychotropic medications can negatively impact on cardiovascular and metabolic health (De Hert *et al.* 2012). Obesity and metabolic disturbances are primary risk factors for CVD and type II diabetes, and are two- to threefold more common in people with mental disorders compared with the general population (Scott & Happell, 2011). Major modifiable risk factors for chronic disease, such as smoking (Lawrence *et al.* 2009), poor diet and physical inactivity (Kilbourne *et al.* 2007; Shatenstein *et al.* 2007) and substance abuse (Scott & Happell, 2011), are overrepresented in people with mental disorders and these may be consequences of symptoms of MNSDs, medication effects and poor emotional regulation (Scott *et al.* 2013).

Interestingly, while schizophrenia was the condition among these mental disorder among for which YLLs were attributed, the number of YLLs were very small compared with the excess mortality associated with the disorder. Our finding of high excess mortality in people with schizophrenia is in line with that found in the previous research (Laursen, 2011; Crump *et al.* 2013; Lawrence *et al.* 2013). Data linkage studies have shown that the majority of deaths in people with schizophrenia are due to chronic disease with CVD accounting for more than one-third of all premature deaths, while unnatural causes, including suicide, homicide and accidents account for just under 15% of excess deaths (Crump *et al.* 2013; Lawrence *et al.* 2013). Despite concerns over the side-effects of antipsychotic medication, lack of antipsychotic treatment has been linked with higher all-cause mortality rates (HR 1.45, 95% confidence interval (CI) 1.20–1.76), with highest risks attributed to cancer (HR 1.94, 95% CI 1.13–3.32) and suicide (HR 2.07, 95% CI 0.73–5.87; Crump *et al.* 2013). Poly-pharmacy and discontinuation of medication also appear to increase risk of all-cause death (Joukamaa *et al.* 2006; Haukka *et al.* 2008).

Research from the UK suggests that the excess mortality rate in schizophrenia and bipolar disorder are comparable (Chang *et al.* 2011). In a recent study, it was estimated that about 80% of premature death in people with bipolar disorder is due to physical disease, almost half of which is explained by CVD (Westman *et al.* 2013). Just under 20% of premature deaths were explained by unnatural causes (suicide, homicide and unintentional injuries; Westman *et al.* 2013).

People with developmental disorders are at twice the risk of premature death compared with the general population (Mouridsen *et al.* 2008). Elevated death rates in autistic spectrum disorders (ASD) are due to several causes, including accidents, respiratory diseases and seizures (Shavelle *et al.* 2001; Mouridsen *et al.* 2008). The elevated mortality risk associated with ASD may be due more to the presence of comorbid medical conditions, particularly epilepsy, and intellectual disability rather than ASD itself (Lee *et al.* 2008; Bilder *et al.* 2013).

Individuals with intellectual disability are expected to have, on average, a life expectancy of 7–12 years less than the general community and life expectancy is dramatically lower in those more severe disability and those with a genetic disorder (e.g., Down syndrome) (Bittles *et al.* 2002). Intellectual disability is associated with greater tendency towards obesity and physical inactivity compared with the general population, and enhanced predisposition to mental disorders, osteoporosis, thyroid disorders, non-ischaemic heart disease and early onset of dementia (Bittles *et al.* 2002). In HIC, causes of death in people with intellectual disability are generally coded under congenital abnormalities, diseases of the nervous system and sense organs, mental disorders and respiratory disease (Tyrer & McGrother, 2009). Information on causes of death in LMIC populations is sparse.

Children with ADHD or CD are two to three times more likely to experience unintentional injuries requiring medical attention compared with children without behavioural disorders (Rowe *et al.* 2004; Lee *et al.* 2008). The injuries most commonly reported included burns, poisoning and frac(Rowe *et al.* 2004). Adolescents and young adults with inattention disorders are more likely to be involved in traffic accidents (Jerome *et al.* 2006). Adults who were identified with behavioural disorders in childhood are at higher risk of cigarette smoking, binge-drinking (ADHD) and obesity (CD) (von Stumm *et al.* 2011) in later life. Despite the strong evidence for an association between childhood behavioural disorders and poorer health outcomes, there is insufficient information available to model the natural history of disease and thus no estimates quantifying excess mortality in this group at population level.

Another important disorder demonstrating an apparent absence of excess-mortality in GBD 2010 is the umbrella anxiety disorders group. This was a necessary choice as the information on excess mortality in anxiety disorders was found to be inconsistent with some anxiety disorders; however, severe presentations such as post-traumatic stress disorder (PTSD), have previously been associated with increased deaths caused by ischaemic heart disease (IHD), neoplasms and intentional and unintentional injuries (Ahmadi *et al.* 2011; Lawrence *et al.* 2013).

While light-to-moderate alcohol consumption has been associated with lower rates of some disease such as diabetes mellitus and coronary heart disease, heavy consumption has been associated with increased rates of chronic disease, including cancer, MNSDs, cardiovascular disease, liver and pancreas diseases (Rehm *et al.* 2010*a*). There is evidence for alcohol as a carcinogen in humans, with particularly strong causal links established between alcoholic beverage consumption and oral cavity, pharynx, larynx, oesophagus, liver, colorectal and female breast cancers (Rehm *et al.* 2010*a*). A consistent relationship has also been found between heavy alcohol consumption and epilepsy (Rehm *et al.* 2010*a*) and it is also implicated in development of depression and personality disorders, although the direction of causality and effect of confounding factors remains uncertain (Rao *et al.* 2000; Rohde *et al.* 2001). Risk of diabetes mellitus, hypertension, stroke, sudden cardiac death and other cardiovascular outcomes is elevated in those with alcohol use disorders (Rehm *et al.* 2010*a*). The relationship between alcohol consumption and liver cirrhosis is well recognised, but alcohol use disorders appear more strongly related to cirrhosis mortality *v.* morbidity as it negatively affects the course of existing liver disease (Rehm *et al.* 2010*b*). Heavy alcohol use is also related to higher rates of infectious diseases, such as tuberculosis, and unintentional and intentional injury, with strong evidence for a dose–response relationship (Rehm *et al.* 2010*a*).

Excess and premature deaths in illicit drug users occur in several ways. Most obvious is the acute toxic effects of illicit drug use which may lead to overdose – the cause-specific deaths generally captured by the ICD-coding system. In addition, a substantial number of deaths are likely due to the more indirect effects of intoxication resulting in accidental injuries and violence. There are a plethora of adverse health outcomes with elevated risks of premature mortality for which illicit drug dependence is an important contributor. These outcomes are often chronic and include cardiovascular disease, liver disease and a range of mental disorders including psychosis. Suicide is an important outcome, particularly for opioid users where an SMR of approximately 14 has been reported in two separate reviews (Degenhardt *et al.* 2011; Chesney *et al.* 2014). Injection of drugs, most common in opioid dependence, carries a high risk of blood-borne bacterial and viral infections, notably HIV, Hepatitis B and Hepatitis C (Mathers *et al.* 2010; Nelson *et al.* 2011).

Epilepsy is associated with two- to threefold higher than mortality in the general community, particularly in childhood onset epilepsy, with the highest standardised mortality ratio encountered in the first year or two after diagnosis (Preux & Druet-Cabanac, 2005; Sillanpaa & Shinnar, 2010; Neligan *et al.* 2010; Trinka *et al.* 2013). Common causes of premature mortality in epilepsy include acute symptomatic disorders (e.g., brain tumour and stroke), sudden unexpected death, suicide and accidents (Hitiris *et al.* 2007). Roughly 85% of people with epilepsy live in LMICs and here the risk of premature mortality is highest (Carpio *et al.* 2005; Diop *et al.* 2005; Newton & Garcia, 2012; Jette & Trevathan, 2014) from status epilepticus, drowning and burns associated with poor access to and/or compliance with medical treatment, cognitive impairment and age (Jilek & Rwiza, 1992; Kamgno *et al.* 2003; Mu *et al.* 2011; Ngugi *et al.* 2014).

As with mental disorders, excess mortality in dementia has been associated with functional disability leading to lifestyle factors (e.g., poor eating behaviours, physical inactivity and poor hygiene) and comorbid or underlying physical conditions, including cardiovascular disease, diabetes mellitus and neoplasms (Guehne *et al.* 2005; Llibre Rodríguez *et al.* 2008). Infections, particularly pneumonia and the complications of urinary tract infections, frequently lead to death in people with dementia (Mitchell *et al.* 2009).

Strategies for reducing mortality associated with MNSDs are primarily related to preventing onset of disorders, reducing case fatality, and preventing onset of fatal sequela. There is growing evidence that excess mortality in people in mental and substance use disorders can be reduced through existing evidence-based treatments and improved screening and treatment for chronic disease. There is some evidence that collaborative care by community-based health teams has the potential to reduce overall death as well as suicide deaths (Malone *et al.* 2007; Dieterich *et al.* 2010). The use of collaborative care models to improve physical health in people with MNSDs is growing in developed countries and these have demonstrated a range of positive health outcomes including reduced cardiovascular risk profiles (Druss *et al.* 2010). The effectiveness of these strategies in preventing premature mortality in LMIC populations has yet to be tested but this may be a cost-effective approach to treatment where trained mental health clinicians are scarce.

To improve life expectancy in people with comorbid mental and physical health issues requires proactive screening and adequate care for chronic disease. Screening and prevention of metabolic risk factors is essential. Strategies for early cancer detection should be prioritised and models of care developed to ensure that people with MNSDs receive the same level of physical health care and treatment as the rest of the population.

Psychiatric treatments, specifically pharmacotherapies, may have some protective effect against excess mortality (Weinmann *et al.* 2009) although evidence suggests that this depends on use of medications according to best practice guidelines (Cullen *et al.* 2013). In contrast, some second generation antipsychotics may actually pose an elevated risk mediated by metabolic side effects (Newcomer, 2005; Smith *et al.* 2008; Rummel-Kluge *et al.* 2010).

Much of the disease burden due to opioid dependence and injecting drug use could be averted by scaling up needle and syringe programs (NSPs), opioid substitution treatment and HIV antiretroviral therapy (Degenhardt *et al.* 2010; Turner *et al.* 2011). Both methadone and buprenorphine (the two most commonly used medications) have been listed on the WHO's *List of Essential Medicines* (World Health Organization, 2005) as core medications for the treatment of opioid dependence (Mattick *et al.* 2008, 2009). OST reduces mortality among opioid-dependent people (Davoli *et al.* 1994; Caplehorn & Drummer, 1999; Brugal *et al.* 2005; Darke *et al.* 2006; Gibson *et al.* 2008; Degenhardt *et al.* 2009*b*), with time spent in treatment halving mortality compared with that in time spent out of treatment (Degenhardt *et al.* 2011). A large evaluation study in multiple countries, including LMICs, has demonstrated that OST is effective in reducing opioid use and injecting risk behaviours and improving physical and mental wellbeing (Lawrinson *et al.* 2008). There is increasing evidence that not only HIV (Degenhardt *et al.* 2010) but also HCV (Turner *et al.* 2011) burden can been reduced through NSPs; HCV burden can also be decreased by effectively treating chronic HCV (Turner *et al.* 2011). The release of more effective and less toxic HCV drugs is expected to dramatically improve what have been extremely low rates of HCV treatment uptake by people who inject drugs (Swan, 2011).

There is also scope for reducing the risk of overdose among people who continue to use opioids. There is increasing evidence that the provision of the opioid antagonist naloxone to opioid users enables peers to effectively intervene if overdoses occur (Galea *et al.* 2006; Sporer & Kral, 2007). Additional strategies may include: education of users about the risks of overdose (especially high risk periods such as post-release from prison or after a period of abstinence), and motivational interviews with users who have recently overdosed (Sporer, 2003). Safe injecting rooms have been proposed as an additional strategy to reduce overdose, although their population reach is likely to be more limited (Hall & Kimber, 2005). There is evidence that psychosocial interventions including self-help programmes and cognitive behavioural therapy are effective in psychostimulant dependence (Baker *et al.* 2005; Knapp *et al.* 2007).

In low-income regions, mortality in epilepsy patients is largely due to preventable causes (Diop *et al.* 2005; Jette & Trevathan, 2014). Yet, the treatment gap is more than 75% in low-income countries, and more than 50% in many lower and upper middle-income countries (Jette & Trevathan, 2014). Legislation to ensure availability of affordable and efficacious anti-seizure medications, clinician education in prescribing antiepileptic medications, and patient education regarding the importance of medical adherence is critical to alleviate the epilepsy treatment gap. Cost-effective epilepsy treatments are available and accurate diagnosis can be made without costly technical equipment. Targeting epilepsy risk factors, including more common structural and metabolic causes of epilepsy will likely decrease mortality risk as well. In addition, education and information provision on safe lifestyle habits in epilepsy patients (i.e., avoiding fires, swimming and driving in those with active convulsive epilepsy) will clearly be beneficial. Education to dispel myths associated with epilepsy among employers and teachers may empower those with epilepsy to seek treatment.

Mortality in dementia patients is commonly by preventable medical conditions, including infections. Caregiver education and support services regarding proper care of patients with cognitive decline will likely decrease infection rates and thus, mortality. Government financial support for healthcare services and caregiver support would also benefit this population. Strategies to enhance nutrition, as well as monitoring and treatment of vascular risk factors including high blood pressure, hypercholesterolemia, smoking, obesity and diabetes, are important measures as well.

### Limitations of the study

Quantifying mortality presents several challenges. Cause of death data is affected by multiple factors, including: certification skills among physicians, diagnostic and other data available for completing the death certificate, cultural variations in choosing and prioritising the cause of death, and institutional parameters for governing mortality reporting (Lozano *et al.* 2012). In LMIC populations, where many deaths are not medically certified, different data sources and diagnostic approaches are used (e.g., from surveillance systems, psychological autopsy work and disease registries) to derive cause of death estimates (Lozano *et al.* 2012). The implication is that cause of death assessments are subject to uncertainty; a good illustration is the widely debated difference in maternal death estimates by GBD and by the United Nations (Byass, 2010).

Cause of death data also provided estimates of deaths due to MNSDs that were not captured within the main GBD 2010 categories. The decision was taken to create residual categories to reflect the additional mortality not captured within specific disorders. Deaths and YLLs were calculated for: ‘other’ mental disorders (16 140 deaths equating to 5.4 YLLs per 100 000 persons); ‘other’ drug use disorders (33 ,561 deaths equating to 22.5 YLLs per 100 000 persons); and ‘other’ neurological disorders (481 142 deaths and 231.6 YLLs per 100 000). The modelling strategies for these residual groups do not allow calculation of excess mortality for comparison as done throughout this paper however the YLL estimates for these groups are exceptionally high.

Mortality directly related to MNSDs is particularly difficult to capture in cause of death data due to the complex web of causality which link them with other physical disorders. Thus it becomes very important to identify and quantify the not inconsiderable excess premature mortality in people with MNSDs through understanding the pathway between these disorders and fatal sequelae.

Although valuable, the CRAs undertaken as part of GBD 2010 provide an incomplete picture. There are almost certainly deaths where we may not have enough information to parse out what is causally related or what is due to confounding. Assuming multiple risk factors are independent of each other is also a limitation as done in CRA methodology. A more accurate quantification of the joint effects of multiple risk factors, that is what explains the difference between excess and cause-specific deaths, is an important area for future research.

## Conclusion

Despite the challenges in quantifying causal mortality in MNSDs it is abundantly clear that the mortality-associated disease burden of mental, substance use and neurological disorders is significant. The continuing life expectancy gap in persons with these disorders represents a lack of parity between this portion of the population and the community in general (Thornicroft, 2013). People with MNSDs face additional barriers to physical health care because of stigma within the healthcare system, the ‘silo’ effect between mental and physical health care caused by overspecialisation, and diagnostic overshadowing of physical health issues by presence of mental disorders (Bailey *et al.* 2013). Differential access to ‘usual’ care for this group leads to poorer outcomes in terms of health loss and mortality and incurs high costs in health care provision (Centre for Mental Health, 2010). Further research and development of new strategies for reducing mortality associated with MNSDs is needed.

## Supplementary material

For supplementary material accompanying this paper visit http://dx.doi.org/10.1017/S2045796014000687. click here to view supplementary material
