# Supplementary material for: Excess mortality from mental, neurological and substance use disorders in the Global Burden of Disease Study 2010
Source: Epidemiol Psychiatr Sci. 2014 Dec 15;24(2):121–40. doi: 10.1017/S2045796014000687 (PMC6998140; doi:10.1017/S2045796014000687)
Supplement: Supplementary file 1 [file S2045796014000687sup001.docx]

Supplementary material

Figure 1: Generic disease model (adapted from diagram presented on page 104 of “The Global Burden of Diseases, Injuries, and Risk Factors Study: Operations Manual ([Institute for Health Metrics and Evaluation, 2009](#_ENREF_1))”.

Figure 2: Age-standardised YLL rates (per 100,000) by disorder as proportion of global YLLs for mental, neurological and substance use disorders in 2010

Table: Cause-specific YLLs as a percentage of all cause YLLs, 2010 (%)

| Region | Cause |  |  |  |  |  |  |  |  |  |  |  |  |  |  |  |  |  |  |  |
| --- | --- | --- | --- | --- | --- | --- | --- | --- | --- | --- | --- | --- | --- | --- | --- | --- | --- | --- | --- | --- |
| Males |  | Age-standardised | 0-1 years | 1-4 years | 5-9 years | 10-14 years | 15-19 years | 20-24 years | 25-29 years | 30-34 years | 35-39 years | 40-44 years | 45-49 years | 50-54 years | 55-59 years | 60-64 years | 65-69 years | 70-74 years | 75-79 years | 80+ years |
| Global | Dementias | 0.232 | 0.000 | 0.000 | 0.194 | 0.149 | 0.071 | 0.052 | 0.047 | 0.057 | 0.071 | 0.078 | 0.100 | 0.102 | 0.127 | 0.193 | 0.287 | 0.497 | 0.909 | 2.090 |
| Asia Pacific, High Income | Dementias | 0.390 | 0.000 | 0.000 | 0.323 | 0.206 | 0.064 | 0.046 | 0.047 | 0.060 | 0.057 | 0.068 | 0.072 | 0.144 | 0.225 | 0.317 | 0.429 | 0.594 | 0.816 | 1.262 |
| Australasia | Dementias | 1.387 | 0.000 | 0.000 | 1.127 | 0.803 | 0.124 | 0.104 | 0.071 | 0.079 | 0.092 | 0.091 | 0.149 | 0.231 | 0.364 | 0.783 | 1.197 | 2.187 | 3.438 | 5.880 |
| Europe, Western | Dementias | 1.080 | 0.000 | 0.000 | 1.217 | 0.739 | 0.216 | 0.130 | 0.096 | 0.103 | 0.092 | 0.117 | 0.128 | 0.202 | 0.324 | 0.562 | 0.947 | 1.575 | 2.675 | 4.319 |
| Latin America, Southern | Dementias | 0.558 | 0.000 | 0.000 | 0.133 | 0.138 | 0.068 | 0.045 | 0.043 | 0.051 | 0.066 | 0.089 | 0.105 | 0.169 | 0.214 | 0.353 | 0.595 | 1.013 | 1.641 | 2.865 |
| North America, High Income | Dementias | 1.399 | 0.000 | 0.000 | 0.735 | 0.475 | 0.078 | 0.047 | 0.041 | 0.049 | 0.063 | 0.085 | 0.117 | 0.210 | 0.407 | 0.752 | 1.302 | 2.485 | 4.259 | 7.875 |
| Asia, Central | Dementias | 0.063 | 0.000 | 0.000 | 0.172 | 0.222 | 0.074 | 0.069 | 0.078 | 0.074 | 0.070 | 0.053 | 0.058 | 0.087 | 0.108 | 0.068 | 0.055 | 0.071 | 0.060 | 0.122 |
| Europe, Central | Dementias | 0.236 | 0.000 | 0.000 | 0.646 | 0.466 | 0.179 | 0.082 | 0.090 | 0.094 | 0.078 | 0.088 | 0.097 | 0.123 | 0.145 | 0.199 | 0.287 | 0.380 | 0.539 | 0.644 |
| Europe, Eastern | Dementias | 0.228 | 0.000 | 0.000 | 0.706 | 0.709 | 0.126 | 0.093 | 0.088 | 0.173 | 0.246 | 0.316 | 0.391 | 0.318 | 0.261 | 0.252 | 0.183 | 0.179 | 0.198 | 0.201 |
| Sub-Saharan Africa, Central | Dementias | 0.042 | 0.000 | 0.000 | 0.142 | 0.102 | 0.043 | 0.035 | 0.033 | 0.035 | 0.038 | 0.038 | 0.046 | 0.024 | 0.035 | 0.050 | 0.070 | 0.111 | 0.179 | 0.308 |
| Sub-Saharan Africa, East | Dementias | 0.060 | 0.000 | 0.000 | 0.139 | 0.095 | 0.048 | 0.042 | 0.041 | 0.043 | 0.042 | 0.042 | 0.050 | 0.036 | 0.052 | 0.067 | 0.096 | 0.152 | 0.239 | 0.426 |
| Sub-Saharan Africa, Southern | Dementias | 0.137 | 0.000 | 0.000 | 0.035 | 0.025 | 0.036 | 0.023 | 0.027 | 0.032 | 0.031 | 0.040 | 0.048 | 0.108 | 0.136 | 0.205 | 0.330 | 0.556 | 0.908 | 1.458 |
| Sub-Saharan Africa, West | Dementias | 0.063 | 0.000 | 0.000 | 0.169 | 0.118 | 0.048 | 0.038 | 0.037 | 0.038 | 0.039 | 0.038 | 0.049 | 0.047 | 0.069 | 0.103 | 0.153 | 0.234 | 0.343 | 0.588 |
| North Africa / Middle East | Dementias | 0.165 | 0.000 | 0.000 | 0.197 | 0.195 | 0.065 | 0.049 | 0.053 | 0.052 | 0.052 | 0.047 | 0.042 | 0.060 | 0.083 | 0.107 | 0.175 | 0.291 | 0.491 | 0.982 |
| Asia, South | Dementias | 0.051 | 0.000 | 0.000 | 0.210 | 0.167 | 0.052 | 0.041 | 0.040 | 0.045 | 0.052 | 0.056 | 0.064 | 0.025 | 0.031 | 0.042 | 0.054 | 0.088 | 0.120 | 0.208 |
| Asia, East | Dementias | 0.265 | 0.000 | 0.000 | 0.224 | 0.196 | 0.222 | 0.114 | 0.081 | 0.090 | 0.119 | 0.087 | 0.117 | 0.130 | 0.142 | 0.193 | 0.249 | 0.406 | 0.598 | 1.063 |
| Asia, Southeast | Dementias | 0.066 | 0.000 | 0.000 | 0.097 | 0.065 | 0.030 | 0.044 | 0.045 | 0.050 | 0.055 | 0.058 | 0.054 | 0.049 | 0.046 | 0.054 | 0.080 | 0.101 | 0.183 | 0.276 |
| Oceania | Dementias | 0.208 | 0.000 | 0.000 | 0.295 | 0.246 | 0.091 | 0.078 | 0.074 | 0.084 | 0.093 | 0.104 | 0.107 | 0.096 | 0.132 | 0.191 | 0.266 | 0.427 | 0.673 | 1.316 |
| Caribbean | Dementias | 0.194 | 0.000 | 0.000 | 0.424 | 0.056 | 0.014 | 0.027 | 0.024 | 0.026 | 0.053 | 0.057 | 0.064 | 0.064 | 0.132 | 0.241 | 0.442 | 0.872 | 1.511 | 3.299 |
| Latin America, Andean | Dementias | 0.082 | 0.000 | 0.000 | 0.133 | 0.079 | 0.041 | 0.026 | 0.030 | 0.056 | 0.041 | 0.042 | 0.055 | 0.056 | 0.056 | 0.090 | 0.113 | 0.216 | 0.277 | 0.396 |
| Latin America, Central | Dementias | 0.129 | 0.000 | 0.000 | 0.167 | 0.122 | 0.036 | 0.025 | 0.031 | 0.036 | 0.043 | 0.063 | 0.064 | 0.089 | 0.101 | 0.143 | 0.199 | 0.278 | 0.431 | 0.619 |
| Latin America, Tropical | Dementias | 0.264 | 0.000 | 0.000 | 0.159 | 0.116 | 0.042 | 0.028 | 0.028 | 0.041 | 0.061 | 0.076 | 0.077 | 0.104 | 0.147 | 0.242 | 0.362 | 0.667 | 1.043 | 1.548 |
| Global | Epilepsy | 0.474 | 0.139 | 0.896 | 1.324 | 1.433 | 1.472 | 1.325 | 1.122 | 0.902 | 0.727 | 0.590 | 0.465 | 0.345 | 0.242 | 0.184 | 0.159 | 0.129 | 0.116 | 0.121 |
| Asia Pacific, High Income | Epilepsy | 0.290 | 0.180 | 1.450 | 1.870 | 2.417 | 1.330 | 1.021 | 0.953 | 0.725 | 0.547 | 0.398 | 0.296 | 0.180 | 0.095 | 0.079 | 0.072 | 0.073 | 0.086 | 0.105 |
| Australasia | Epilepsy | 0.598 | 0.080 | 1.576 | 2.473 | 2.107 | 1.341 | 1.615 | 1.687 | 1.758 | 1.469 | 1.343 | 0.898 | 0.436 | 0.310 | 0.171 | 0.134 | 0.126 | 0.104 | 0.093 |
| Europe, Western | Epilepsy | 0.499 | 0.126 | 1.881 | 1.987 | 2.168 | 1.231 | 1.264 | 1.366 | 1.409 | 1.267 | 1.038 | 0.743 | 0.483 | 0.322 | 0.223 | 0.170 | 0.143 | 0.132 | 0.110 |
| Latin America, Southern | Epilepsy | 0.307 | 0.079 | 1.098 | 1.052 | 0.979 | 0.762 | 0.745 | 0.950 | 0.761 | 0.781 | 0.569 | 0.391 | 0.285 | 0.156 | 0.111 | 0.086 | 0.078 | 0.063 | 0.063 |
| North America, High Income | Epilepsy | 0.207 | 0.040 | 0.618 | 0.807 | 0.713 | 0.372 | 0.422 | 0.486 | 0.502 | 0.446 | 0.352 | 0.251 | 0.181 | 0.115 | 0.083 | 0.074 | 0.067 | 0.073 | 0.092 |
| Asia, Central | Epilepsy | 0.700 | 0.049 | 1.142 | 2.852 | 4.124 | 4.068 | 3.053 | 2.232 | 1.577 | 1.256 | 0.832 | 0.554 | 0.321 | 0.204 | 0.112 | 0.066 | 0.042 | 0.025 | 0.020 |
| Europe, Central | Epilepsy | 0.434 | 0.156 | 1.487 | 2.486 | 2.290 | 1.303 | 1.026 | 1.194 | 1.261 | 1.234 | 0.968 | 0.693 | 0.453 | 0.255 | 0.148 | 0.102 | 0.067 | 0.047 | 0.033 |
| Europe, Eastern | Epilepsy | 0.302 | 0.044 | 0.773 | 1.533 | 1.710 | 1.215 | 0.820 | 0.615 | 0.541 | 0.526 | 0.454 | 0.350 | 0.229 | 0.139 | 0.080 | 0.041 | 0.021 | 0.012 | 0.006 |
| Sub-Saharan Africa, Central | Epilepsy | 0.738 | 0.149 | 0.695 | 1.395 | 1.917 | 2.262 | 2.067 | 1.645 | 1.354 | 1.170 | 1.007 | 0.839 | 0.701 | 0.562 | 0.421 | 0.327 | 0.277 | 0.240 | 0.241 |
| Sub-Saharan Africa, East | Epilepsy | 1.197 | 0.219 | 0.812 | 1.964 | 2.049 | 3.861 | 2.987 | 2.484 | 1.970 | 1.698 | 1.601 | 1.477 | 1.355 | 1.087 | 0.893 | 0.588 | 0.501 | 0.441 | 0.451 |
| Sub-Saharan Africa, Southern | Epilepsy | 0.779 | 0.085 | 0.394 | 0.724 | 0.973 | 1.530 | 1.119 | 0.814 | 0.732 | 0.754 | 0.867 | 1.105 | 1.303 | 1.303 | 1.132 | 0.850 | 0.691 | 0.561 | 0.445 |
| Sub-Saharan Africa, West | Epilepsy | 1.205 | 0.234 | 1.242 | 2.435 | 3.164 | 3.869 | 3.382 | 2.730 | 2.157 | 1.881 | 1.638 | 1.473 | 1.282 | 0.998 | 0.785 | 0.625 | 0.523 | 0.438 | 0.484 |
| North Africa / Middle East | Epilepsy | 0.288 | 0.097 | 0.815 | 1.234 | 1.243 | 1.057 | 1.007 | 0.795 | 0.834 | 0.530 | 0.434 | 0.269 | 0.170 | 0.110 | 0.082 | 0.068 | 0.067 | 0.055 | 0.058 |
| Asia, South | Epilepsy | 0.255 | 0.070 | 0.519 | 0.628 | 0.779 | 0.460 | 0.577 | 0.494 | 0.358 | 0.294 | 0.290 | 0.276 | 0.226 | 0.194 | 0.166 | 0.210 | 0.202 | 0.216 | 0.318 |
| Asia, East | Epilepsy | 0.286 | 0.109 | 0.711 | 1.036 | 1.421 | 1.479 | 1.246 | 0.938 | 0.729 | 0.517 | 0.376 | 0.250 | 0.186 | 0.114 | 0.073 | 0.055 | 0.039 | 0.035 | 0.033 |
| Asia, Southeast | Epilepsy | 0.417 | 0.142 | 0.844 | 1.228 | 1.829 | 1.364 | 1.187 | 0.983 | 0.733 | 0.640 | 0.563 | 0.408 | 0.274 | 0.217 | 0.152 | 0.131 | 0.113 | 0.091 | 0.099 |
| Oceania | Epilepsy | 0.618 | 0.125 | 0.980 | 1.911 | 2.881 | 2.071 | 1.786 | 1.673 | 1.228 | 0.948 | 1.050 | 0.584 | 0.433 | 0.306 | 0.222 | 0.176 | 0.148 | 0.137 | 0.147 |
| Caribbean | Epilepsy | 0.167 | 0.088 | 0.285 | 0.419 | 0.080 | 0.148 | 0.160 | 0.151 | 0.173 | 0.253 | 0.259 | 0.248 | 0.237 | 0.181 | 0.143 | 0.135 | 0.122 | 0.109 | 0.082 |
| Latin America, Andean | Epilepsy | 0.538 | 0.094 | 0.821 | 1.405 | 2.188 | 1.967 | 1.364 | 1.090 | 1.094 | 0.872 | 0.762 | 0.641 | 0.455 | 0.358 | 0.256 | 0.198 | 0.125 | 0.135 | 0.084 |
| Latin America, Central | Epilepsy | 0.480 | 0.162 | 1.292 | 1.559 | 1.777 | 1.104 | 0.827 | 0.815 | 0.769 | 0.767 | 0.639 | 0.481 | 0.372 | 0.286 | 0.214 | 0.159 | 0.137 | 0.120 | 0.105 |
| Latin America, Tropical | Epilepsy | 0.307 | 0.101 | 1.075 | 1.025 | 1.029 | 0.501 | 0.421 | 0.517 | 0.614 | 0.630 | 0.564 | 0.442 | 0.297 | 0.202 | 0.144 | 0.098 | 0.086 | 0.076 | 0.070 |
| Global | Schizophrenia | 0.000 | 0.000 | 0.000 | 0.000 | 0.000 | 0.000 | 0.000 | 0.178 | 0.242 | 0.185 | 0.122 | 0.106 | 0.078 | 0.077 | 0.046 | 0.034 | 0.027 | 0.017 | 0.014 |
| Asia Pacific, High Income | Schizophrenia | 0.000 | 0.000 | 0.000 | 0.000 | 0.000 | 0.000 | 0.000 | 0.663 | 1.177 | 0.690 | 0.429 | 0.491 | 0.411 | 0.273 | 0.155 | 0.084 | 0.055 | 0.027 | 0.011 |
| Australasia | Schizophrenia | 0.001 | 0.000 | 0.000 | 0.000 | 0.000 | 0.000 | 0.000 | 0.210 | 0.278 | 0.085 | 0.054 | 0.043 | 0.061 | 0.047 | 0.041 | 0.034 | 0.032 | 0.032 | 0.038 |
| Europe, Western | Schizophrenia | 0.000 | 0.000 | 0.000 | 0.000 | 0.000 | 0.000 | 0.000 | 0.421 | 0.471 | 0.214 | 0.189 | 0.230 | 0.235 | 0.158 | 0.104 | 0.057 | 0.045 | 0.030 | 0.026 |
| Latin America, Southern | Schizophrenia | 0.001 | 0.000 | 0.000 | 0.000 | 0.000 | 0.000 | 0.000 | 0.140 | 0.099 | 0.065 | 0.055 | 0.063 | 0.059 | 0.068 | 0.054 | 0.029 | 0.020 | 0.009 | 0.005 |
| North America, High Income | Schizophrenia | 0.000 | 0.000 | 0.000 | 0.000 | 0.000 | 0.000 | 0.000 | 0.148 | 0.217 | 0.053 | 0.048 | 0.069 | 0.093 | 0.070 | 0.050 | 0.032 | 0.022 | 0.017 | 0.021 |
| Asia, Central | Schizophrenia | 0.007 | 0.000 | 0.000 | 0.000 | 0.000 | 0.000 | 0.000 | 1.299 | 0.970 | 0.483 | 0.246 | 0.254 | 0.205 | 0.092 | 0.035 | 0.014 | 0.013 | 0.009 | 0.003 |
| Europe, Central | Schizophrenia | 0.000 | 0.000 | 0.000 | 0.000 | 0.000 | 0.000 | 0.000 | 0.472 | 0.550 | 0.208 | 0.187 | 0.170 | 0.185 | 0.135 | 0.070 | 0.036 | 0.016 | 0.007 | 0.005 |
| Europe, Eastern | Schizophrenia | 0.000 | 0.000 | 0.000 | 0.000 | 0.000 | 0.000 | 0.000 | 0.599 | 0.600 | 0.135 | 0.058 | 0.043 | 0.035 | 0.031 | 0.018 | 0.007 | 0.005 | 0.002 | 0.001 |
| Sub-Saharan Africa, Central | Schizophrenia | 0.004 | 0.000 | 0.000 | 0.000 | 0.000 | 0.000 | 0.000 | 0.074 | 0.141 | 0.106 | 0.055 | 0.049 | 0.038 | 0.036 | 0.025 | 0.023 | 0.013 | 0.006 | 0.002 |
| Sub-Saharan Africa, East | Schizophrenia | 0.001 | 0.000 | 0.000 | 0.000 | 0.000 | 0.000 | 0.000 | 0.118 | 0.208 | 0.114 | 0.053 | 0.038 | 0.029 | 0.029 | 0.022 | 0.021 | 0.014 | 0.008 | 0.004 |
| Sub-Saharan Africa, Southern | Schizophrenia | 0.008 | 0.000 | 0.000 | 0.000 | 0.000 | 0.000 | 0.000 | 0.387 | 0.574 | 0.182 | 0.072 | 0.042 | 0.039 | 0.040 | 0.037 | 0.045 | 0.044 | 0.033 | 0.025 |
| Sub-Saharan Africa, West | Schizophrenia | 0.001 | 0.000 | 0.000 | 0.000 | 0.000 | 0.000 | 0.000 | 0.055 | 0.125 | 0.095 | 0.043 | 0.035 | 0.027 | 0.027 | 0.025 | 0.026 | 0.017 | 0.009 | 0.004 |
| North Africa / Middle East | Schizophrenia | 0.000 | 0.000 | 0.000 | 0.000 | 0.000 | 0.000 | 0.000 | 0.092 | 0.267 | 0.099 | 0.048 | 0.083 | 0.039 | 0.127 | 0.026 | 0.017 | 0.011 | 0.009 | 0.009 |
| Asia, South | Schizophrenia | 0.000 | 0.000 | 0.000 | 0.000 | 0.000 | 0.000 | 0.000 | 0.023 | 0.036 | 0.093 | 0.068 | 0.069 | 0.016 | 0.015 | 0.009 | 0.027 | 0.022 | 0.019 | 0.022 |
| Asia, East | Schizophrenia | 0.000 | 0.000 | 0.000 | 0.000 | 0.000 | 0.000 | 0.000 | 1.707 | 1.596 | 0.800 | 0.514 | 0.508 | 0.397 | 0.298 | 0.140 | 0.079 | 0.059 | 0.026 | 0.015 |
| Asia, Southeast | Schizophrenia | 0.000 | 0.000 | 0.000 | 0.000 | 0.000 | 0.000 | 0.000 | 0.290 | 0.274 | 0.177 | 0.115 | 0.062 | 0.082 | 0.038 | 0.021 | 0.015 | 0.014 | 0.009 | 0.006 |
| Oceania | Schizophrenia | 0.036 | 0.000 | 0.000 | 0.000 | 0.000 | 0.000 | 0.000 | 0.324 | 0.394 | 0.283 | 0.178 | 0.140 | 0.094 | 0.062 | 0.035 | 0.026 | 0.013 | 0.007 | 0.003 |
| Caribbean | Schizophrenia | 0.004 | 0.000 | 0.000 | 0.000 | 0.000 | 0.000 | 0.000 | 0.081 | 0.020 | 0.022 | 0.019 | 0.040 | 0.043 | 0.053 | 0.034 | 0.046 | 0.040 | 0.022 | 0.015 |
| Latin America, Andean | Schizophrenia | 0.001 | 0.000 | 0.000 | 0.000 | 0.000 | 0.000 | 0.000 | 0.040 | 0.102 | 0.024 | 0.019 | 0.020 | 0.015 | 0.016 | 0.011 | 0.015 | 0.005 | 0.005 | 0.003 |
| Latin America, Central | Schizophrenia | 0.000 | 0.000 | 0.000 | 0.000 | 0.000 | 0.000 | 0.000 | 0.099 | 0.073 | 0.029 | 0.021 | 0.021 | 0.021 | 0.020 | 0.018 | 0.015 | 0.009 | 0.006 | 0.004 |
| Latin America, Tropical | Schizophrenia | 0.000 | 0.000 | 0.000 | 0.000 | 0.000 | 0.000 | 0.000 | 0.196 | 0.191 | 0.052 | 0.050 | 0.062 | 0.057 | 0.053 | 0.028 | 0.014 | 0.015 | 0.006 | 0.005 |
| Global | Alcohol | 0.000 | 0.000 | 0.000 | 0.000 | 0.000 | 0.010 | 0.077 | 0.736 | 1.597 | 1.191 | 1.086 | 1.253 | 1.203 | 0.876 | 0.510 | 0.249 | 0.135 | 0.057 | 0.028 |
| Asia Pacific, High Income | Alcohol | 0.002 | 0.000 | 0.000 | 0.000 | 0.000 | 0.069 | 0.452 | 1.631 | 3.493 | 2.841 | 3.107 | 3.243 | 2.602 | 1.364 | 0.846 | 0.403 | 0.175 | 0.080 | 0.031 |
| Australasia | Alcohol | 0.034 | 0.000 | 0.000 | 0.000 | 0.000 | 0.150 | 0.835 | 4.476 | 5.133 | 2.321 | 2.184 | 2.440 | 2.525 | 1.867 | 1.589 | 0.756 | 0.482 | 0.263 | 0.139 |
| Europe, Western | Alcohol | 0.004 | 0.000 | 0.000 | 0.000 | 0.000 | 0.090 | 1.621 | 5.920 | 10.956 | 6.478 | 7.926 | 10.656 | 10.087 | 6.182 | 3.105 | 1.284 | 0.622 | 0.249 | 0.101 |
| Latin America, Southern | Alcohol | 0.028 | 0.000 | 0.000 | 0.000 | 0.000 | 0.037 | 0.443 | 1.861 | 3.341 | 1.948 | 2.309 | 3.340 | 3.495 | 2.909 | 2.070 | 1.141 | 0.517 | 0.206 | 0.092 |
| North America, High Income | Alcohol | 0.005 | 0.000 | 0.000 | 0.000 | 0.000 | 0.198 | 2.060 | 6.521 | 7.304 | 2.705 | 3.245 | 5.135 | 5.759 | 3.946 | 1.978 | 0.752 | 0.295 | 0.147 | 0.077 |
| Asia, Central | Alcohol | 0.042 | 0.000 | 0.000 | 0.000 | 0.000 | 0.025 | 0.364 | 2.493 | 3.507 | 2.075 | 1.565 | 2.043 | 2.076 | 1.084 | 0.480 | 0.139 | 0.107 | 0.030 | 0.016 |
| Europe, Central | Alcohol | 0.022 | 0.000 | 0.000 | 0.000 | 0.000 | 0.183 | 3.561 | 14.336 | 27.579 | 14.519 | 11.467 | 13.865 | 12.069 | 6.913 | 2.902 | 0.957 | 0.342 | 0.123 | 0.036 |
| Europe, Eastern | Alcohol | 0.033 | 0.000 | 0.000 | 0.000 | 0.000 | 0.233 | 5.652 | 30.928 | 58.176 | 20.844 | 9.660 | 8.101 | 7.411 | 5.381 | 2.539 | 0.683 | 0.372 | 0.104 | 0.042 |
| Sub-Saharan Africa, Central | Alcohol | 0.003 | 0.000 | 0.000 | 0.000 | 0.000 | 0.000 | 0.002 | 0.032 | 0.080 | 0.066 | 0.047 | 0.044 | 0.042 | 0.034 | 0.027 | 0.016 | 0.009 | 0.004 | 0.001 |
| Sub-Saharan Africa, East | Alcohol | 0.001 | 0.000 | 0.000 | 0.000 | 0.000 | 0.001 | 0.004 | 0.030 | 0.051 | 0.032 | 0.023 | 0.025 | 0.021 | 0.023 | 0.026 | 0.019 | 0.015 | 0.009 | 0.004 |
| Sub-Saharan Africa, Southern | Alcohol | 0.018 | 0.000 | 0.000 | 0.000 | 0.000 | 0.010 | 0.068 | 0.468 | 0.858 | 0.379 | 0.177 | 0.118 | 0.120 | 0.132 | 0.104 | 0.100 | 0.061 | 0.035 | 0.014 |
| Sub-Saharan Africa, West | Alcohol | 0.000 | 0.000 | 0.000 | 0.000 | 0.000 | 0.000 | 0.000 | 0.005 | 0.013 | 0.011 | 0.006 | 0.006 | 0.006 | 0.006 | 0.007 | 0.006 | 0.004 | 0.003 | 0.001 |
| North Africa / Middle East | Alcohol | 0.000 | 0.000 | 0.000 | 0.000 | 0.000 | 0.002 | 0.032 | 0.146 | 0.217 | 0.133 | 0.077 | 0.083 | 0.064 | 0.038 | 0.037 | 0.014 | 0.012 | 0.020 | 0.003 |
| Asia, South | Alcohol | 0.000 | 0.000 | 0.000 | 0.000 | 0.000 | 0.006 | 0.056 | 0.217 | 0.360 | 0.289 | 0.258 | 0.268 | 0.251 | 0.185 | 0.089 | 0.054 | 0.033 | 0.017 | 0.009 |
| Asia, East | Alcohol | 0.000 | 0.000 | 0.000 | 0.000 | 0.000 | 0.026 | 0.303 | 0.807 | 1.041 | 0.677 | 0.527 | 0.542 | 0.460 | 0.329 | 0.129 | 0.073 | 0.035 | 0.016 | 0.008 |
| Asia, Southeast | Alcohol | 0.002 | 0.000 | 0.000 | 0.000 | 0.000 | 0.010 | 0.108 | 0.952 | 1.694 | 1.168 | 0.877 | 0.835 | 0.612 | 0.388 | 0.230 | 0.103 | 0.047 | 0.023 | 0.014 |
| Oceania | Alcohol | 0.111 | 0.000 | 0.000 | 0.000 | 0.000 | 0.007 | 0.055 | 0.571 | 1.005 | 0.714 | 0.656 | 0.478 | 0.373 | 0.212 | 0.133 | 0.068 | 0.030 | 0.012 | 0.004 |
| Caribbean | Alcohol | 0.030 | 0.000 | 0.000 | 0.000 | 0.000 | 0.018 | 0.073 | 0.322 | 0.101 | 0.148 | 0.280 | 0.334 | 0.427 | 0.475 | 0.321 | 0.297 | 0.184 | 0.082 | 0.045 |
| Latin America, Andean | Alcohol | 0.062 | 0.000 | 0.000 | 0.000 | 0.000 | 0.032 | 0.276 | 1.381 | 2.210 | 1.632 | 1.302 | 1.503 | 1.512 | 1.538 | 1.337 | 0.831 | 0.561 | 0.231 | 0.089 |
| Latin America, Central | Alcohol | 0.020 | 0.000 | 0.000 | 0.000 | 0.000 | 0.075 | 0.684 | 4.385 | 5.936 | 2.366 | 1.887 | 2.202 | 2.056 | 1.796 | 1.187 | 0.811 | 0.533 | 0.291 | 0.186 |
| Latin America, Tropical | Alcohol | 0.023 | 0.000 | 0.000 | 0.000 | 0.000 | 0.041 | 1.459 | 7.420 | 11.217 | 3.792 | 3.307 | 3.540 | 3.134 | 2.214 | 1.267 | 0.625 | 0.319 | 0.139 | 0.092 |
| Global | Opioid | 0.143 | 0.028 | 0.059 | 0.072 | 0.072 | 0.217 | 0.460 | 0.569 | 0.493 | 0.365 | 0.314 | 0.239 | 0.150 | 0.080 | 0.046 | 0.029 | 0.024 | 0.021 | 0.019 |
| Asia Pacific, High Income | Opioid | 0.068 | 0.032 | 0.083 | 0.060 | 0.067 | 0.108 | 0.171 | 0.272 | 0.267 | 0.227 | 0.150 | 0.091 | 0.052 | 0.035 | 0.027 | 0.023 | 0.020 | 0.019 | 0.017 |
| Australasia | Opioid | 1.290 | 0.091 | 0.471 | 0.319 | 0.545 | 2.155 | 4.931 | 7.082 | 6.616 | 4.355 | 2.400 | 1.350 | 0.540 | 0.224 | 0.101 | 0.057 | 0.042 | 0.030 | 0.021 |
| Europe, Western | Opioid | 0.852 | 0.058 | 0.237 | 0.197 | 0.494 | 2.028 | 4.452 | 6.142 | 5.380 | 3.274 | 1.670 | 0.686 | 0.268 | 0.114 | 0.059 | 0.036 | 0.027 | 0.020 | 0.017 |
| Latin America, Southern | Opioid | 0.132 | 0.158 | 0.483 | 0.275 | 0.195 | 0.218 | 0.252 | 0.229 | 0.237 | 0.201 | 0.154 | 0.120 | 0.096 | 0.087 | 0.086 | 0.079 | 0.077 | 0.077 | 0.072 |
| North America, High Income | Opioid | 1.714 | 0.030 | 0.404 | 0.220 | 0.459 | 1.977 | 4.169 | 6.055 | 6.518 | 5.778 | 4.716 | 2.927 | 1.540 | 0.659 | 0.256 | 0.111 | 0.066 | 0.045 | 0.033 |
| Asia, Central | Opioid | 0.341 | 0.122 | 0.409 | 0.651 | 0.759 | 0.529 | 1.084 | 1.389 | 1.233 | 0.800 | 0.491 | 0.319 | 0.147 | 0.127 | 0.068 | 0.054 | 0.108 | 0.084 | 0.056 |
| Europe, Central | Opioid | 0.205 | 0.171 | 0.636 | 0.508 | 0.367 | 0.725 | 1.515 | 1.634 | 0.930 | 0.394 | 0.227 | 0.134 | 0.087 | 0.059 | 0.037 | 0.030 | 0.022 | 0.017 | 0.011 |
| Europe, Eastern | Opioid | 0.666 | 0.209 | 0.873 | 1.051 | 0.812 | 2.012 | 3.170 | 2.716 | 1.802 | 0.979 | 0.596 | 0.404 | 0.270 | 0.175 | 0.140 | 0.080 | 0.063 | 0.062 | 0.030 |
| Sub-Saharan Africa, Central | Opioid | 0.028 | 0.012 | 0.024 | 0.021 | 0.020 | 0.054 | 0.084 | 0.080 | 0.064 | 0.049 | 0.040 | 0.031 | 0.026 | 0.016 | 0.011 | 0.009 | 0.008 | 0.007 | 0.007 |
| Sub-Saharan Africa, East | Opioid | 0.091 | 0.022 | 0.087 | 0.068 | 0.058 | 0.176 | 0.296 | 0.276 | 0.213 | 0.141 | 0.112 | 0.082 | 0.069 | 0.050 | 0.033 | 0.027 | 0.023 | 0.021 | 0.019 |
| Sub-Saharan Africa, Southern | Opioid | 0.361 | 0.671 | 0.412 | 0.492 | 0.404 | 0.567 | 0.445 | 0.331 | 0.341 | 0.238 | 0.276 | 0.313 | 0.351 | 0.361 | 0.218 | 0.171 | 0.191 | 0.168 | 0.174 |
| Sub-Saharan Africa, West | Opioid | 0.030 | 0.011 | 0.027 | 0.027 | 0.025 | 0.068 | 0.105 | 0.100 | 0.075 | 0.051 | 0.039 | 0.031 | 0.026 | 0.019 | 0.014 | 0.012 | 0.010 | 0.009 | 0.007 |
| North Africa / Middle East | Opioid | 0.305 | 0.069 | 0.158 | 0.205 | 0.190 | 0.334 | 0.792 | 1.282 | 1.138 | 0.882 | 0.750 | 0.585 | 0.391 | 0.251 | 0.134 | 0.106 | 0.077 | 0.078 | 0.082 |
| Asia, South | Opioid | 0.013 | 0.004 | 0.015 | 0.016 | 0.015 | 0.030 | 0.048 | 0.053 | 0.046 | 0.032 | 0.024 | 0.016 | 0.011 | 0.007 | 0.005 | 0.004 | 0.003 | 0.003 | 0.002 |
| Asia, East | Opioid | 0.024 | 0.009 | 0.024 | 0.039 | 0.029 | 0.045 | 0.079 | 0.132 | 0.116 | 0.081 | 0.048 | 0.024 | 0.013 | 0.006 | 0.004 | 0.003 | 0.002 | 0.002 | 0.003 |
| Asia, Southeast | Opioid | 0.044 | 0.006 | 0.045 | 0.048 | 0.037 | 0.066 | 0.127 | 0.138 | 0.124 | 0.094 | 0.102 | 0.048 | 0.037 | 0.019 | 0.014 | 0.015 | 0.010 | 0.008 | 0.010 |
| Oceania | Opioid | 0.081 | 0.026 | 0.111 | 0.126 | 0.088 | 0.165 | 0.157 | 0.257 | 0.214 | 0.145 | 0.096 | 0.074 | 0.058 | 0.045 | 0.039 | 0.035 | 0.067 | 0.034 | 0.038 |
| Caribbean | Opioid | 0.064 | 0.129 | 0.183 | 0.168 | 0.026 | 0.030 | 0.047 | 0.044 | 0.041 | 0.057 | 0.046 | 0.061 | 0.057 | 0.067 | 0.047 | 0.035 | 0.027 | 0.043 | 0.030 |
| Latin America, Andean | Opioid | 0.304 | 0.086 | 0.649 | 0.749 | 0.538 | 0.911 | 0.770 | 0.541 | 0.514 | 0.373 | 0.390 | 0.295 | 0.284 | 0.218 | 0.189 | 0.174 | 0.124 | 0.122 | 0.087 |
| Latin America, Central | Opioid | 0.142 | 0.053 | 0.246 | 0.161 | 0.130 | 0.182 | 0.239 | 0.265 | 0.302 | 0.303 | 0.231 | 0.178 | 0.129 | 0.091 | 0.067 | 0.051 | 0.051 | 0.048 | 0.043 |
| Latin America, Tropical | Opioid | 0.069 | 0.024 | 0.216 | 0.110 | 0.089 | 0.123 | 0.122 | 0.157 | 0.146 | 0.110 | 0.087 | 0.071 | 0.054 | 0.047 | 0.038 | 0.035 | 0.033 | 0.035 | 0.034 |
| Global | Cocaine | 0.002 | 0.000 | 0.001 | 0.001 | 0.001 | 0.003 | 0.006 | 0.007 | 0.006 | 0.004 | 0.004 | 0.003 | 0.002 | 0.001 | 0.001 | 0.000 | 0.000 | 0.000 | 0.000 |
| Asia Pacific, High Income | Cocaine | 0.001 | 0.001 | 0.002 | 0.001 | 0.001 | 0.002 | 0.003 | 0.005 | 0.005 | 0.004 | 0.003 | 0.002 | 0.001 | 0.001 | 0.000 | 0.000 | 0.000 | 0.000 | 0.000 |
| Australasia | Cocaine | 0.028 | 0.002 | 0.014 | 0.009 | 0.013 | 0.057 | 0.106 | 0.144 | 0.132 | 0.095 | 0.054 | 0.029 | 0.012 | 0.005 | 0.003 | 0.001 | 0.001 | 0.001 | 0.001 |
| Europe, Western | Cocaine | 0.008 | 0.001 | 0.004 | 0.002 | 0.006 | 0.023 | 0.044 | 0.057 | 0.046 | 0.027 | 0.013 | 0.005 | 0.002 | 0.001 | 0.001 | 0.000 | 0.000 | 0.000 | 0.000 |
| Latin America, Southern | Cocaine | 0.002 | 0.003 | 0.009 | 0.005 | 0.003 | 0.004 | 0.005 | 0.004 | 0.005 | 0.003 | 0.003 | 0.002 | 0.002 | 0.002 | 0.001 | 0.001 | 0.002 | 0.001 | 0.001 |
| North America, High Income | Cocaine | 0.026 | 0.001 | 0.009 | 0.004 | 0.007 | 0.030 | 0.065 | 0.089 | 0.112 | 0.083 | 0.076 | 0.043 | 0.021 | 0.010 | 0.004 | 0.002 | 0.001 | 0.001 | 0.001 |
| Asia, Central | Cocaine | 0.004 | 0.001 | 0.005 | 0.008 | 0.009 | 0.007 | 0.015 | 0.017 | 0.016 | 0.009 | 0.006 | 0.004 | 0.002 | 0.002 | 0.001 | 0.001 | 0.001 | 0.001 | 0.001 |
| Europe, Central | Cocaine | 0.002 | 0.002 | 0.006 | 0.005 | 0.004 | 0.007 | 0.015 | 0.013 | 0.008 | 0.003 | 0.002 | 0.001 | 0.001 | 0.001 | 0.000 | 0.000 | 0.000 | 0.000 | 0.000 |
| Europe, Eastern | Cocaine | 0.008 | 0.003 | 0.014 | 0.016 | 0.011 | 0.024 | 0.038 | 0.029 | 0.021 | 0.010 | 0.007 | 0.005 | 0.003 | 0.002 | 0.002 | 0.001 | 0.001 | 0.001 | 0.000 |
| Sub-Saharan Africa, Central | Cocaine | 0.000 | 0.000 | 0.000 | 0.000 | 0.000 | 0.000 | 0.000 | 0.000 | 0.000 | 0.000 | 0.000 | 0.000 | 0.000 | 0.000 | 0.000 | 0.000 | 0.000 | 0.000 | 0.000 |
| Sub-Saharan Africa, East | Cocaine | 0.000 | 0.000 | 0.000 | 0.000 | 0.000 | 0.001 | 0.001 | 0.001 | 0.001 | 0.001 | 0.001 | 0.000 | 0.001 | 0.000 | 0.000 | 0.000 | 0.000 | 0.000 | 0.000 |
| Sub-Saharan Africa, Southern | Cocaine | 0.005 | 0.008 | 0.007 | 0.009 | 0.008 | 0.007 | 0.005 | 0.003 | 0.005 | 0.003 | 0.003 | 0.005 | 0.006 | 0.005 | 0.003 | 0.002 | 0.004 | 0.004 | 0.004 |
| Sub-Saharan Africa, West | Cocaine | 0.000 | 0.000 | 0.000 | 0.000 | 0.000 | 0.001 | 0.001 | 0.001 | 0.001 | 0.000 | 0.000 | 0.000 | 0.000 | 0.000 | 0.000 | 0.000 | 0.000 | 0.000 | 0.000 |
| North Africa / Middle East | Cocaine | 0.004 | 0.001 | 0.003 | 0.003 | 0.003 | 0.005 | 0.013 | 0.018 | 0.019 | 0.011 | 0.009 | 0.008 | 0.005 | 0.003 | 0.002 | 0.001 | 0.001 | 0.001 | 0.001 |
| Asia, South | Cocaine | 0.000 | 0.000 | 0.000 | 0.000 | 0.000 | 0.000 | 0.000 | 0.000 | 0.000 | 0.000 | 0.000 | 0.000 | 0.000 | 0.000 | 0.000 | 0.000 | 0.000 | 0.000 | 0.000 |
| Asia, East | Cocaine | 0.000 | 0.000 | 0.001 | 0.001 | 0.001 | 0.001 | 0.002 | 0.002 | 0.002 | 0.001 | 0.001 | 0.000 | 0.000 | 0.000 | 0.000 | 0.000 | 0.000 | 0.000 | 0.000 |
| Asia, Southeast | Cocaine | 0.001 | 0.000 | 0.001 | 0.001 | 0.001 | 0.001 | 0.002 | 0.002 | 0.002 | 0.001 | 0.002 | 0.001 | 0.001 | 0.000 | 0.000 | 0.000 | 0.000 | 0.000 | 0.000 |
| Oceania | Cocaine | 0.002 | 0.000 | 0.002 | 0.002 | 0.002 | 0.003 | 0.004 | 0.004 | 0.004 | 0.002 | 0.002 | 0.002 | 0.001 | 0.001 | 0.001 | 0.001 | 0.001 | 0.001 | 0.001 |
| Caribbean | Cocaine | 0.001 | 0.001 | 0.003 | 0.002 | 0.000 | 0.000 | 0.000 | 0.000 | 0.000 | 0.001 | 0.000 | 0.001 | 0.001 | 0.001 | 0.001 | 0.000 | 0.000 | 0.000 | 0.000 |
| Latin America, Andean | Cocaine | 0.004 | 0.001 | 0.008 | 0.008 | 0.007 | 0.014 | 0.012 | 0.007 | 0.007 | 0.005 | 0.005 | 0.003 | 0.003 | 0.003 | 0.002 | 0.001 | 0.002 | 0.001 | 0.001 |
| Latin America, Central | Cocaine | 0.003 | 0.001 | 0.007 | 0.003 | 0.003 | 0.004 | 0.005 | 0.005 | 0.006 | 0.005 | 0.004 | 0.004 | 0.003 | 0.002 | 0.001 | 0.001 | 0.001 | 0.001 | 0.001 |
| Latin America, Tropical | Cocaine | 0.001 | 0.000 | 0.004 | 0.002 | 0.001 | 0.002 | 0.001 | 0.003 | 0.002 | 0.001 | 0.001 | 0.001 | 0.001 | 0.001 | 0.001 | 0.001 | 0.001 | 0.001 | 0.000 |
| Global | Amphetamine | 0.002 | 0.000 | 0.001 | 0.001 | 0.001 | 0.002 | 0.005 | 0.006 | 0.006 | 0.004 | 0.003 | 0.002 | 0.002 | 0.001 | 0.000 | 0.000 | 0.000 | 0.000 | 0.000 |
| Asia Pacific, High Income | Amphetamine | 0.001 | 0.001 | 0.001 | 0.001 | 0.001 | 0.002 | 0.003 | 0.005 | 0.005 | 0.003 | 0.003 | 0.001 | 0.001 | 0.000 | 0.000 | 0.000 | 0.000 | 0.000 | 0.000 |
| Australasia | Amphetamine | 0.031 | 0.002 | 0.015 | 0.009 | 0.015 | 0.061 | 0.117 | 0.160 | 0.147 | 0.105 | 0.059 | 0.032 | 0.013 | 0.006 | 0.003 | 0.001 | 0.001 | 0.001 | 0.001 |
| Europe, Western | Amphetamine | 0.010 | 0.001 | 0.004 | 0.003 | 0.007 | 0.028 | 0.054 | 0.071 | 0.057 | 0.034 | 0.017 | 0.006 | 0.003 | 0.001 | 0.001 | 0.000 | 0.000 | 0.000 | 0.000 |
| Latin America, Southern | Amphetamine | 0.002 | 0.003 | 0.009 | 0.005 | 0.003 | 0.004 | 0.004 | 0.004 | 0.004 | 0.003 | 0.003 | 0.002 | 0.001 | 0.002 | 0.001 | 0.001 | 0.001 | 0.001 | 0.001 |
| North America, High Income | Amphetamine | 0.017 | 0.000 | 0.007 | 0.002 | 0.005 | 0.019 | 0.042 | 0.056 | 0.077 | 0.051 | 0.050 | 0.027 | 0.013 | 0.006 | 0.002 | 0.002 | 0.001 | 0.001 | 0.000 |
| Asia, Central | Amphetamine | 0.003 | 0.001 | 0.004 | 0.006 | 0.006 | 0.005 | 0.011 | 0.012 | 0.011 | 0.006 | 0.004 | 0.003 | 0.002 | 0.001 | 0.001 | 0.001 | 0.001 | 0.001 | 0.001 |
| Europe, Central | Amphetamine | 0.002 | 0.002 | 0.006 | 0.005 | 0.004 | 0.007 | 0.015 | 0.013 | 0.008 | 0.003 | 0.002 | 0.001 | 0.001 | 0.001 | 0.000 | 0.000 | 0.000 | 0.000 | 0.000 |
| Europe, Eastern | Amphetamine | 0.005 | 0.002 | 0.010 | 0.011 | 0.008 | 0.016 | 0.024 | 0.017 | 0.013 | 0.006 | 0.004 | 0.003 | 0.002 | 0.001 | 0.001 | 0.001 | 0.001 | 0.001 | 0.000 |
| Sub-Saharan Africa, Central | Amphetamine | 0.000 | 0.000 | 0.000 | 0.000 | 0.000 | 0.000 | 0.000 | 0.000 | 0.000 | 0.000 | 0.000 | 0.000 | 0.000 | 0.000 | 0.000 | 0.000 | 0.000 | 0.000 | 0.000 |
| Sub-Saharan Africa, East | Amphetamine | 0.000 | 0.000 | 0.000 | 0.000 | 0.000 | 0.001 | 0.001 | 0.001 | 0.001 | 0.000 | 0.000 | 0.000 | 0.001 | 0.000 | 0.000 | 0.000 | 0.000 | 0.000 | 0.000 |
| Sub-Saharan Africa, Southern | Amphetamine | 0.005 | 0.008 | 0.007 | 0.008 | 0.008 | 0.006 | 0.005 | 0.003 | 0.005 | 0.003 | 0.003 | 0.005 | 0.006 | 0.005 | 0.003 | 0.002 | 0.003 | 0.003 | 0.004 |
| Sub-Saharan Africa, West | Amphetamine | 0.000 | 0.000 | 0.000 | 0.000 | 0.000 | 0.001 | 0.001 | 0.001 | 0.001 | 0.000 | 0.000 | 0.000 | 0.000 | 0.000 | 0.000 | 0.000 | 0.000 | 0.000 | 0.000 |
| North Africa / Middle East | Amphetamine | 0.006 | 0.001 | 0.003 | 0.004 | 0.004 | 0.007 | 0.016 | 0.024 | 0.024 | 0.014 | 0.012 | 0.010 | 0.007 | 0.004 | 0.002 | 0.002 | 0.001 | 0.001 | 0.001 |
| Asia, South | Amphetamine | 0.000 | 0.000 | 0.000 | 0.000 | 0.000 | 0.000 | 0.000 | 0.000 | 0.000 | 0.000 | 0.000 | 0.000 | 0.000 | 0.000 | 0.000 | 0.000 | 0.000 | 0.000 | 0.000 |
| Asia, East | Amphetamine | 0.000 | 0.000 | 0.001 | 0.001 | 0.001 | 0.001 | 0.002 | 0.002 | 0.002 | 0.001 | 0.001 | 0.000 | 0.000 | 0.000 | 0.000 | 0.000 | 0.000 | 0.000 | 0.000 |
| Asia, Southeast | Amphetamine | 0.001 | 0.000 | 0.001 | 0.001 | 0.001 | 0.001 | 0.002 | 0.002 | 0.002 | 0.001 | 0.002 | 0.001 | 0.001 | 0.000 | 0.000 | 0.000 | 0.000 | 0.000 | 0.000 |
| Oceania | Amphetamine | 0.002 | 0.000 | 0.002 | 0.002 | 0.002 | 0.003 | 0.004 | 0.004 | 0.004 | 0.002 | 0.002 | 0.002 | 0.001 | 0.001 | 0.001 | 0.001 | 0.001 | 0.001 | 0.001 |
| Caribbean | Amphetamine | 0.001 | 0.002 | 0.003 | 0.003 | 0.000 | 0.000 | 0.001 | 0.001 | 0.001 | 0.001 | 0.001 | 0.001 | 0.001 | 0.001 | 0.001 | 0.001 | 0.000 | 0.001 | 0.000 |
| Latin America, Andean | Amphetamine | 0.004 | 0.001 | 0.008 | 0.008 | 0.007 | 0.015 | 0.012 | 0.008 | 0.007 | 0.006 | 0.005 | 0.004 | 0.003 | 0.003 | 0.002 | 0.002 | 0.002 | 0.002 | 0.001 |
| Latin America, Central | Amphetamine | 0.002 | 0.001 | 0.005 | 0.002 | 0.002 | 0.003 | 0.003 | 0.003 | 0.003 | 0.003 | 0.003 | 0.002 | 0.002 | 0.001 | 0.001 | 0.001 | 0.001 | 0.001 | 0.001 |
| Latin America, Tropical | Amphetamine | 0.001 | 0.000 | 0.004 | 0.002 | 0.001 | 0.002 | 0.002 | 0.003 | 0.002 | 0.001 | 0.001 | 0.001 | 0.001 | 0.001 | 0.001 | 0.001 | 0.001 | 0.001 | 0.000 |
| Females |  | Age-standardised | 0-1 years | 1-4 years | 5-9 years | 10-14 years | 15-19 years | 20-24 years | 25-29 years | 30-34 years | 35-39 years | 40-44 years | 45-49 years | 50-54 years | 55-59 years | 60-64 years | 65-69 years | 70-74 years | 75-79 years | 80+ years |
| Global | Dementias | 0.320 | 0.000 | 0.000 | 0.190 | 0.186 | 0.096 | 0.074 | 0.050 | 0.052 | 0.068 | 0.084 | 0.090 | 0.118 | 0.180 | 0.266 | 0.383 | 0.632 | 1.231 | 3.236 |
| Asia Pacific, High Income | Dementias | 0.666 | 0.000 | 0.000 | 0.428 | 0.298 | 0.106 | 0.079 | 0.068 | 0.066 | 0.080 | 0.101 | 0.093 | 0.154 | 0.306 | 0.467 | 0.677 | 0.978 | 1.465 | 2.300 |
| Australasia | Dementias | 2.122 | 0.000 | 0.000 | 1.765 | 1.222 | 0.211 | 0.180 | 0.119 | 0.138 | 0.116 | 0.127 | 0.153 | 0.284 | 0.604 | 1.115 | 1.607 | 2.750 | 4.493 | 8.895 |
| Europe, Western | Dementias | 1.793 | 0.000 | 0.000 | 1.487 | 1.105 | 0.387 | 0.285 | 0.222 | 0.182 | 0.149 | 0.160 | 0.177 | 0.259 | 0.502 | 0.879 | 1.400 | 2.323 | 3.992 | 6.566 |
| Latin America, Southern | Dementias | 0.858 | 0.000 | 0.000 | 0.174 | 0.173 | 0.106 | 0.064 | 0.086 | 0.070 | 0.075 | 0.109 | 0.102 | 0.164 | 0.276 | 0.500 | 0.837 | 1.478 | 2.575 | 4.370 |
| North America, High Income | Dementias | 2.271 | 0.000 | 0.000 | 0.792 | 0.558 | 0.145 | 0.105 | 0.076 | 0.089 | 0.095 | 0.102 | 0.172 | 0.298 | 0.592 | 1.014 | 1.701 | 3.254 | 5.855 | 12.007 |
| Asia, Central | Dementias | 0.070 | 0.000 | 0.000 | 0.252 | 0.247 | 0.150 | 0.169 | 0.156 | 0.114 | 0.074 | 0.120 | 0.108 | 0.090 | 0.090 | 0.108 | 0.061 | 0.051 | 0.053 | 0.063 |
| Europe, Central | Dementias | 0.402 | 0.000 | 0.000 | 1.098 | 0.708 | 0.307 | 0.167 | 0.154 | 0.147 | 0.132 | 0.109 | 0.110 | 0.153 | 0.241 | 0.389 | 0.451 | 0.619 | 0.804 | 0.863 |
| Europe, Eastern | Dementias | 0.263 | 0.000 | 0.000 | 1.332 | 1.160 | 0.271 | 0.207 | 0.100 | 0.165 | 0.290 | 0.325 | 0.335 | 0.369 | 0.343 | 0.345 | 0.247 | 0.245 | 0.244 | 0.214 |
| Sub-Saharan Africa, Central | Dementias | 0.058 | 0.000 | 0.000 | 0.109 | 0.097 | 0.049 | 0.040 | 0.025 | 0.025 | 0.028 | 0.029 | 0.033 | 0.036 | 0.063 | 0.093 | 0.121 | 0.171 | 0.272 | 0.509 |
| Sub-Saharan Africa, East | Dementias | 0.077 | 0.000 | 0.000 | 0.112 | 0.099 | 0.065 | 0.050 | 0.031 | 0.030 | 0.032 | 0.038 | 0.042 | 0.041 | 0.068 | 0.103 | 0.132 | 0.193 | 0.322 | 0.633 |
| Sub-Saharan Africa, Southern | Dementias | 0.136 | 0.000 | 0.000 | 0.041 | 0.061 | 0.047 | 0.047 | 0.036 | 0.031 | 0.035 | 0.037 | 0.041 | 0.055 | 0.120 | 0.199 | 0.464 | 0.560 | 0.895 | 1.658 |
| Sub-Saharan Africa, West | Dementias | 0.078 | 0.000 | 0.000 | 0.134 | 0.114 | 0.061 | 0.050 | 0.031 | 0.029 | 0.032 | 0.037 | 0.043 | 0.047 | 0.088 | 0.133 | 0.183 | 0.258 | 0.419 | 0.811 |
| North Africa / Middle East | Dementias | 0.203 | 0.000 | 0.000 | 0.275 | 0.293 | 0.206 | 0.087 | 0.076 | 0.090 | 0.070 | 0.078 | 0.063 | 0.079 | 0.163 | 0.147 | 0.244 | 0.353 | 0.517 | 0.963 |
| Asia, South | Dementias | 0.086 | 0.000 | 0.000 | 0.199 | 0.195 | 0.079 | 0.069 | 0.051 | 0.053 | 0.063 | 0.070 | 0.068 | 0.042 | 0.065 | 0.092 | 0.110 | 0.161 | 0.253 | 0.436 |
| Asia, East | Dementias | 0.412 | 0.000 | 0.000 | 0.358 | 0.399 | 0.321 | 0.202 | 0.127 | 0.108 | 0.117 | 0.149 | 0.117 | 0.182 | 0.233 | 0.265 | 0.365 | 0.527 | 0.879 | 1.645 |
| Asia, Southeast | Dementias | 0.090 | 0.000 | 0.000 | 0.071 | 0.091 | 0.051 | 0.032 | 0.027 | 0.028 | 0.038 | 0.040 | 0.032 | 0.040 | 0.045 | 0.087 | 0.119 | 0.170 | 0.221 | 0.455 |
| Oceania | Dementias | 0.211 | 0.000 | 0.000 | 0.306 | 0.304 | 0.135 | 0.113 | 0.074 | 0.079 | 0.095 | 0.090 | 0.083 | 0.089 | 0.138 | 0.217 | 0.258 | 0.399 | 0.619 | 1.245 |
| Caribbean | Dementias | 0.339 | 0.000 | 0.000 | 0.306 | 0.069 | 0.053 | 0.174 | 0.045 | 0.055 | 0.166 | 0.051 | 0.066 | 0.069 | 0.184 | 0.339 | 0.637 | 1.223 | 2.016 | 4.415 |
| Latin America, Andean | Dementias | 0.112 | 0.000 | 0.000 | 0.158 | 0.127 | 0.064 | 0.064 | 0.044 | 0.070 | 0.057 | 0.041 | 0.070 | 0.066 | 0.068 | 0.109 | 0.151 | 0.221 | 0.342 | 0.550 |
| Latin America, Central | Dementias | 0.192 | 0.000 | 0.000 | 0.187 | 0.158 | 0.071 | 0.047 | 0.042 | 0.044 | 0.042 | 0.054 | 0.061 | 0.090 | 0.106 | 0.160 | 0.224 | 0.374 | 0.595 | 0.883 |
| Latin America, Tropical | Dementias | 0.421 | 0.000 | 0.000 | 0.181 | 0.164 | 0.082 | 0.069 | 0.064 | 0.049 | 0.047 | 0.068 | 0.058 | 0.093 | 0.157 | 0.291 | 0.444 | 0.774 | 1.393 | 2.266 |
| Global | Epilepsy | 0.531 | 0.167 | 1.138 | 1.448 | 1.502 | 1.496 | 1.339 | 1.036 | 0.875 | 0.777 | 0.652 | 0.485 | 0.360 | 0.275 | 0.199 | 0.216 | 0.154 | 0.148 | 0.132 |
| Asia Pacific, High Income | Epilepsy | 0.383 | 0.239 | 1.640 | 1.893 | 2.681 | 1.940 | 1.597 | 1.287 | 0.874 | 0.616 | 0.415 | 0.283 | 0.205 | 0.122 | 0.077 | 0.080 | 0.070 | 0.075 | 0.092 |
| Australasia | Epilepsy | 0.577 | 0.079 | 1.984 | 2.666 | 2.175 | 2.067 | 2.635 | 2.170 | 1.983 | 1.375 | 0.958 | 0.616 | 0.399 | 0.246 | 0.213 | 0.169 | 0.133 | 0.121 | 0.086 |
| Europe, Western | Epilepsy | 0.505 | 0.144 | 2.115 | 2.169 | 2.216 | 2.230 | 2.483 | 1.964 | 1.491 | 1.136 | 0.744 | 0.580 | 0.388 | 0.296 | 0.236 | 0.186 | 0.184 | 0.163 | 0.121 |
| Latin America, Southern | Epilepsy | 0.321 | 0.079 | 1.020 | 1.310 | 1.293 | 1.419 | 1.281 | 1.296 | 0.984 | 0.743 | 0.443 | 0.340 | 0.200 | 0.150 | 0.087 | 0.073 | 0.082 | 0.060 | 0.057 |
| North America, High Income | Epilepsy | 0.229 | 0.040 | 0.655 | 0.905 | 0.923 | 0.710 | 0.879 | 0.817 | 0.633 | 0.493 | 0.353 | 0.253 | 0.188 | 0.134 | 0.108 | 0.078 | 0.073 | 0.075 | 0.073 |
| Asia, Central | Epilepsy | 0.870 | 0.054 | 1.024 | 3.434 | 4.891 | 5.632 | 4.541 | 3.611 | 2.761 | 2.026 | 1.251 | 0.740 | 0.353 | 0.201 | 0.106 | 0.063 | 0.035 | 0.026 | 0.017 |
| Europe, Central | Epilepsy | 0.347 | 0.123 | 1.581 | 2.355 | 2.974 | 1.917 | 1.985 | 1.723 | 1.301 | 0.946 | 0.577 | 0.392 | 0.269 | 0.174 | 0.123 | 0.078 | 0.049 | 0.040 | 0.023 |
| Europe, Eastern | Epilepsy | 0.354 | 0.040 | 0.734 | 1.927 | 2.080 | 2.061 | 1.805 | 1.323 | 0.921 | 0.640 | 0.472 | 0.373 | 0.251 | 0.140 | 0.077 | 0.035 | 0.017 | 0.009 | 0.004 |
| Sub-Saharan Africa, Central | Epilepsy | 0.480 | 0.122 | 0.639 | 1.105 | 1.459 | 1.506 | 1.133 | 0.784 | 0.659 | 0.630 | 0.524 | 0.501 | 0.461 | 0.343 | 0.266 | 0.211 | 0.165 | 0.146 | 0.155 |
| Sub-Saharan Africa, East | Epilepsy | 0.968 | 0.257 | 1.232 | 1.957 | 1.867 | 1.976 | 1.623 | 1.224 | 1.067 | 0.937 | 1.075 | 1.216 | 1.376 | 1.672 | 0.841 | 0.682 | 0.556 | 0.506 | 0.559 |
| Sub-Saharan Africa, Southern | Epilepsy | 0.502 | 0.066 | 0.280 | 0.639 | 1.245 | 1.651 | 0.839 | 0.438 | 0.414 | 0.519 | 0.512 | 0.620 | 0.700 | 0.697 | 0.553 | 0.538 | 0.441 | 0.391 | 0.300 |
| Sub-Saharan Africa, West | Epilepsy | 1.323 | 0.297 | 1.764 | 3.070 | 3.726 | 4.191 | 3.114 | 2.232 | 1.810 | 1.630 | 1.411 | 1.335 | 1.157 | 0.910 | 0.700 | 0.577 | 0.443 | 0.396 | 0.594 |
| North Africa / Middle East | Epilepsy | 0.353 | 0.120 | 1.005 | 1.482 | 1.582 | 1.533 | 1.450 | 1.161 | 1.058 | 0.743 | 0.494 | 0.353 | 0.236 | 0.152 | 0.095 | 0.085 | 0.067 | 0.059 | 0.069 |
| Asia, South | Epilepsy | 0.307 | 0.107 | 0.538 | 0.638 | 0.730 | 0.547 | 0.552 | 0.360 | 0.335 | 0.502 | 0.488 | 0.274 | 0.230 | 0.160 | 0.141 | 0.326 | 0.251 | 0.348 | 0.506 |
| Asia, East | Epilepsy | 0.307 | 0.120 | 0.682 | 1.306 | 1.480 | 1.804 | 1.399 | 1.320 | 0.955 | 0.685 | 0.539 | 0.351 | 0.202 | 0.110 | 0.108 | 0.062 | 0.059 | 0.048 | 0.028 |
| Asia, Southeast | Epilepsy | 0.415 | 0.114 | 0.971 | 1.239 | 1.695 | 1.513 | 1.341 | 1.069 | 0.889 | 0.667 | 0.596 | 0.445 | 0.297 | 0.238 | 0.188 | 0.134 | 0.096 | 0.079 | 0.089 |
| Oceania | Epilepsy | 0.782 | 0.303 | 1.629 | 3.681 | 2.716 | 3.253 | 2.212 | 1.910 | 1.843 | 1.159 | 0.814 | 0.627 | 0.422 | 0.315 | 0.248 | 0.188 | 0.158 | 0.143 | 0.177 |
| Caribbean | Epilepsy | 0.168 | 0.052 | 0.312 | 0.404 | 0.179 | 0.244 | 0.239 | 0.160 | 0.228 | 0.261 | 0.230 | 0.200 | 0.162 | 0.122 | 0.115 | 0.102 | 0.106 | 0.096 | 0.061 |
| Latin America, Andean | Epilepsy | 0.513 | 0.080 | 0.914 | 1.656 | 2.297 | 2.167 | 2.010 | 1.810 | 1.343 | 1.071 | 0.832 | 0.575 | 0.374 | 0.233 | 0.182 | 0.119 | 0.086 | 0.088 | 0.055 |
| Latin America, Central | Epilepsy | 0.558 | 0.172 | 1.319 | 1.941 | 2.098 | 2.053 | 2.006 | 1.815 | 1.533 | 1.226 | 0.875 | 0.567 | 0.356 | 0.249 | 0.204 | 0.155 | 0.128 | 0.112 | 0.075 |
| Latin America, Tropical | Epilepsy | 0.280 | 0.084 | 1.095 | 1.075 | 1.135 | 1.085 | 1.090 | 0.919 | 0.773 | 0.627 | 0.478 | 0.342 | 0.203 | 0.137 | 0.106 | 0.094 | 0.081 | 0.063 | 0.061 |
| Global | Schizophrenia | 0.000 | 0.000 | 0.000 | 0.000 | 0.000 | 0.000 | 0.000 | 0.125 | 0.180 | 0.135 | 0.138 | 0.100 | 0.087 | 0.092 | 0.067 | 0.051 | 0.037 | 0.028 | 0.028 |
| Asia Pacific, High Income | Schizophrenia | 0.001 | 0.000 | 0.000 | 0.000 | 0.000 | 0.000 | 0.000 | 0.921 | 1.364 | 0.727 | 0.567 | 0.635 | 0.452 | 0.253 | 0.231 | 0.157 | 0.101 | 0.061 | 0.051 |
| Australasia | Schizophrenia | 0.002 | 0.000 | 0.000 | 0.000 | 0.000 | 0.000 | 0.000 | 0.149 | 0.253 | 0.136 | 0.154 | 0.093 | 0.121 | 0.070 | 0.063 | 0.046 | 0.050 | 0.062 | 0.102 |
| Europe, Western | Schizophrenia | 0.000 | 0.000 | 0.000 | 0.000 | 0.000 | 0.000 | 0.000 | 0.240 | 0.300 | 0.239 | 0.263 | 0.324 | 0.282 | 0.197 | 0.122 | 0.080 | 0.081 | 0.074 | 0.093 |
| Latin America, Southern | Schizophrenia | 0.001 | 0.000 | 0.000 | 0.000 | 0.000 | 0.000 | 0.000 | 0.092 | 0.080 | 0.103 | 0.115 | 0.135 | 0.097 | 0.103 | 0.083 | 0.037 | 0.036 | 0.018 | 0.016 |
| North America, High Income | Schizophrenia | 0.000 | 0.000 | 0.000 | 0.000 | 0.000 | 0.000 | 0.000 | 0.083 | 0.111 | 0.063 | 0.094 | 0.130 | 0.089 | 0.070 | 0.059 | 0.045 | 0.038 | 0.038 | 0.069 |
| Asia, Central | Schizophrenia | 0.006 | 0.000 | 0.000 | 0.000 | 0.000 | 0.000 | 0.000 | 0.840 | 1.118 | 0.566 | 0.397 | 0.515 | 0.220 | 0.131 | 0.060 | 0.029 | 0.037 | 0.031 | 0.015 |
| Europe, Central | Schizophrenia | 0.001 | 0.000 | 0.000 | 0.000 | 0.000 | 0.000 | 0.000 | 0.388 | 0.555 | 0.341 | 0.465 | 0.498 | 0.414 | 0.325 | 0.162 | 0.101 | 0.059 | 0.033 | 0.019 |
| Europe, Eastern | Schizophrenia | 0.000 | 0.000 | 0.000 | 0.000 | 0.000 | 0.000 | 0.000 | 0.541 | 0.432 | 0.200 | 0.133 | 0.113 | 0.113 | 0.114 | 0.074 | 0.028 | 0.027 | 0.014 | 0.012 |
| Sub-Saharan Africa, Central | Schizophrenia | 0.002 | 0.000 | 0.000 | 0.000 | 0.000 | 0.000 | 0.000 | 0.058 | 0.090 | 0.060 | 0.035 | 0.026 | 0.027 | 0.028 | 0.034 | 0.028 | 0.021 | 0.011 | 0.004 |
| Sub-Saharan Africa, East | Schizophrenia | 0.001 | 0.000 | 0.000 | 0.000 | 0.000 | 0.000 | 0.000 | 0.126 | 0.170 | 0.088 | 0.052 | 0.037 | 0.033 | 0.041 | 0.041 | 0.037 | 0.029 | 0.018 | 0.011 |
| Sub-Saharan Africa, Southern | Schizophrenia | 0.004 | 0.000 | 0.000 | 0.000 | 0.000 | 0.000 | 0.000 | 0.391 | 0.367 | 0.146 | 0.036 | 0.025 | 0.020 | 0.032 | 0.038 | 0.049 | 0.057 | 0.031 | 0.027 |
| Sub-Saharan Africa, West | Schizophrenia | 0.000 | 0.000 | 0.000 | 0.000 | 0.000 | 0.000 | 0.000 | 0.017 | 0.031 | 0.019 | 0.009 | 0.008 | 0.008 | 0.012 | 0.015 | 0.015 | 0.012 | 0.007 | 0.003 |
| North Africa / Middle East | Schizophrenia | 0.000 | 0.000 | 0.000 | 0.000 | 0.000 | 0.000 | 0.000 | 0.079 | 0.162 | 0.097 | 0.259 | 0.074 | 0.033 | 0.055 | 0.036 | 0.018 | 0.011 | 0.009 | 0.007 |
| Asia, South | Schizophrenia | 0.000 | 0.000 | 0.000 | 0.000 | 0.000 | 0.000 | 0.000 | 0.032 | 0.043 | 0.027 | 0.026 | 0.027 | 0.026 | 0.027 | 0.023 | 0.012 | 0.010 | 0.005 | 0.004 |
| Asia, East | Schizophrenia | 0.000 | 0.000 | 0.000 | 0.000 | 0.000 | 0.000 | 0.000 | 1.234 | 1.617 | 1.280 | 1.453 | 1.032 | 0.714 | 0.469 | 0.222 | 0.179 | 0.102 | 0.076 | 0.070 |
| Asia, Southeast | Schizophrenia | 0.000 | 0.000 | 0.000 | 0.000 | 0.000 | 0.000 | 0.000 | 0.160 | 0.197 | 0.120 | 0.105 | 0.078 | 0.070 | 0.050 | 0.028 | 0.017 | 0.012 | 0.011 | 0.008 |
| Oceania | Schizophrenia | 0.091 | 0.000 | 0.000 | 0.000 | 0.000 | 0.000 | 0.000 | 0.886 | 0.871 | 0.688 | 0.534 | 0.432 | 0.266 | 0.154 | 0.157 | 0.102 | 0.064 | 0.038 | 0.021 |
| Caribbean | Schizophrenia | 0.004 | 0.000 | 0.000 | 0.000 | 0.000 | 0.000 | 0.000 | 0.128 | 0.031 | 0.038 | 0.068 | 0.038 | 0.071 | 0.059 | 0.054 | 0.035 | 0.055 | 0.034 | 0.022 |
| Latin America, Andean | Schizophrenia | 0.001 | 0.000 | 0.000 | 0.000 | 0.000 | 0.000 | 0.000 | 0.051 | 0.075 | 0.030 | 0.027 | 0.040 | 0.029 | 0.023 | 0.016 | 0.013 | 0.008 | 0.006 | 0.004 |
| Latin America, Central | Schizophrenia | 0.000 | 0.000 | 0.000 | 0.000 | 0.000 | 0.000 | 0.000 | 0.059 | 0.059 | 0.041 | 0.051 | 0.054 | 0.051 | 0.041 | 0.026 | 0.019 | 0.017 | 0.010 | 0.009 |
| Latin America, Tropical | Schizophrenia | 0.000 | 0.000 | 0.000 | 0.000 | 0.000 | 0.000 | 0.000 | 0.095 | 0.120 | 0.103 | 0.108 | 0.112 | 0.099 | 0.070 | 0.043 | 0.025 | 0.017 | 0.010 | 0.010 |
| Global | Alcohol | 0.000 | 0.000 | 0.000 | 0.000 | 0.000 | 0.005 | 0.018 | 0.153 | 0.300 | 0.292 | 0.312 | 0.359 | 0.416 | 0.357 | 0.228 | 0.121 | 0.073 | 0.032 | 0.021 |
| Asia Pacific, High Income | Alcohol | 0.001 | 0.000 | 0.000 | 0.000 | 0.000 | 0.008 | 0.068 | 0.502 | 0.873 | 0.975 | 0.792 | 0.663 | 0.496 | 0.196 | 0.129 | 0.067 | 0.039 | 0.022 | 0.012 |
| Australasia | Alcohol | 0.014 | 0.000 | 0.000 | 0.000 | 0.000 | 0.057 | 0.252 | 1.463 | 2.093 | 1.195 | 1.635 | 1.839 | 1.548 | 0.843 | 0.529 | 0.250 | 0.153 | 0.084 | 0.066 |
| Europe, Western | Alcohol | 0.002 | 0.000 | 0.000 | 0.000 | 0.000 | 0.024 | 0.302 | 1.601 | 3.805 | 3.876 | 6.256 | 7.780 | 5.985 | 3.208 | 1.495 | 0.590 | 0.310 | 0.136 | 0.082 |
| Latin America, Southern | Alcohol | 0.004 | 0.000 | 0.000 | 0.000 | 0.000 | 0.004 | 0.054 | 0.229 | 0.441 | 0.498 | 0.783 | 0.891 | 0.819 | 0.571 | 0.313 | 0.177 | 0.087 | 0.055 | 0.035 |
| North America, High Income | Alcohol | 0.002 | 0.000 | 0.000 | 0.000 | 0.000 | 0.058 | 0.499 | 2.044 | 3.676 | 2.635 | 3.571 | 4.504 | 3.382 | 1.807 | 0.862 | 0.323 | 0.129 | 0.058 | 0.037 |
| Asia, Central | Alcohol | 0.015 | 0.000 | 0.000 | 0.000 | 0.000 | 0.010 | 0.099 | 0.752 | 1.318 | 1.139 | 1.002 | 1.120 | 1.162 | 0.641 | 0.323 | 0.112 | 0.061 | 0.029 | 0.021 |
| Europe, Central | Alcohol | 0.005 | 0.000 | 0.000 | 0.000 | 0.000 | 0.047 | 0.507 | 2.100 | 4.446 | 4.147 | 5.610 | 6.384 | 4.636 | 2.711 | 1.089 | 0.380 | 0.144 | 0.066 | 0.030 |
| Europe, Eastern | Alcohol | 0.008 | 0.000 | 0.000 | 0.000 | 0.000 | 0.133 | 1.168 | 6.110 | 11.887 | 8.229 | 6.355 | 5.725 | 6.533 | 5.365 | 2.758 | 0.867 | 0.479 | 0.158 | 0.120 |
| Sub-Saharan Africa, Central | Alcohol | 0.002 | 0.000 | 0.000 | 0.000 | 0.000 | 0.000 | 0.002 | 0.019 | 0.045 | 0.043 | 0.029 | 0.023 | 0.030 | 0.027 | 0.024 | 0.020 | 0.009 | 0.004 | 0.001 |
| Sub-Saharan Africa, East | Alcohol | 0.000 | 0.000 | 0.000 | 0.000 | 0.000 | 0.001 | 0.003 | 0.021 | 0.033 | 0.024 | 0.016 | 0.014 | 0.019 | 0.023 | 0.027 | 0.029 | 0.022 | 0.014 | 0.006 |
| Sub-Saharan Africa, Southern | Alcohol | 0.004 | 0.000 | 0.000 | 0.000 | 0.000 | 0.006 | 0.034 | 0.182 | 0.248 | 0.124 | 0.046 | 0.033 | 0.030 | 0.053 | 0.051 | 0.079 | 0.049 | 0.026 | 0.016 |
| Sub-Saharan Africa, West | Alcohol | 0.000 | 0.000 | 0.000 | 0.000 | 0.000 | 0.000 | 0.000 | 0.005 | 0.012 | 0.010 | 0.007 | 0.006 | 0.007 | 0.009 | 0.009 | 0.009 | 0.006 | 0.003 | 0.001 |
| North Africa / Middle East | Alcohol | 0.000 | 0.000 | 0.000 | 0.000 | 0.000 | 0.001 | 0.006 | 0.024 | 0.032 | 0.022 | 0.119 | 0.037 | 0.037 | 0.024 | 0.007 | 0.009 | 0.004 | 0.002 | 0.002 |
| Asia, South | Alcohol | 0.000 | 0.000 | 0.000 | 0.000 | 0.000 | 0.006 | 0.032 | 0.120 | 0.172 | 0.139 | 0.144 | 0.154 | 0.179 | 0.157 | 0.092 | 0.063 | 0.036 | 0.018 | 0.010 |
| Asia, East | Alcohol | 0.000 | 0.000 | 0.000 | 0.000 | 0.000 | 0.024 | 0.025 | 0.205 | 0.153 | 0.115 | 0.148 | 0.111 | 0.065 | 0.047 | 0.024 | 0.012 | 0.012 | 0.004 | 0.005 |
| Asia, Southeast | Alcohol | 0.000 | 0.000 | 0.000 | 0.000 | 0.000 | 0.002 | 0.008 | 0.071 | 0.158 | 0.173 | 0.182 | 0.156 | 0.147 | 0.086 | 0.057 | 0.033 | 0.013 | 0.007 | 0.003 |
| Oceania | Alcohol | 0.017 | 0.000 | 0.000 | 0.000 | 0.000 | 0.002 | 0.009 | 0.103 | 0.135 | 0.157 | 0.126 | 0.076 | 0.069 | 0.031 | 0.024 | 0.016 | 0.006 | 0.003 | 0.001 |
| Caribbean | Alcohol | 0.005 | 0.000 | 0.000 | 0.000 | 0.000 | 0.007 | 0.021 | 0.164 | 0.067 | 0.046 | 0.082 | 0.077 | 0.093 | 0.083 | 0.060 | 0.053 | 0.035 | 0.024 | 0.017 |
| Latin America, Andean | Alcohol | 0.008 | 0.000 | 0.000 | 0.000 | 0.000 | 0.005 | 0.024 | 0.135 | 0.274 | 0.274 | 0.353 | 0.387 | 0.376 | 0.293 | 0.232 | 0.155 | 0.085 | 0.049 | 0.027 |
| Latin America, Central | Alcohol | 0.001 | 0.000 | 0.000 | 0.000 | 0.000 | 0.008 | 0.041 | 0.237 | 0.442 | 0.368 | 0.404 | 0.477 | 0.390 | 0.276 | 0.168 | 0.106 | 0.071 | 0.041 | 0.037 |
| Latin America, Tropical | Alcohol | 0.003 | 0.000 | 0.000 | 0.000 | 0.000 | 0.008 | 0.227 | 1.153 | 1.794 | 1.620 | 1.775 | 1.428 | 0.862 | 0.517 | 0.251 | 0.116 | 0.065 | 0.034 | 0.026 |
| Global | Opioid | 0.074 | 0.022 | 0.065 | 0.054 | 0.071 | 0.150 | 0.193 | 0.206 | 0.180 | 0.165 | 0.175 | 0.148 | 0.108 | 0.063 | 0.037 | 0.026 | 0.023 | 0.018 | 0.016 |
| Asia Pacific, High Income | Opioid | 0.093 | 0.030 | 0.080 | 0.063 | 0.183 | 0.237 | 0.447 | 0.457 | 0.358 | 0.265 | 0.163 | 0.095 | 0.058 | 0.041 | 0.028 | 0.023 | 0.024 | 0.022 | 0.018 |
| Australasia | Opioid | 0.753 | 0.063 | 0.348 | 0.295 | 0.493 | 2.174 | 5.210 | 4.698 | 3.955 | 2.510 | 1.730 | 0.870 | 0.516 | 0.273 | 0.128 | 0.079 | 0.044 | 0.032 | 0.018 |
| Europe, Western | Opioid | 0.322 | 0.046 | 0.148 | 0.142 | 0.407 | 1.581 | 2.814 | 2.823 | 1.950 | 1.158 | 0.632 | 0.309 | 0.151 | 0.091 | 0.050 | 0.032 | 0.025 | 0.018 | 0.014 |
| Latin America, Southern | Opioid | 0.127 | 0.149 | 0.498 | 0.263 | 0.160 | 0.207 | 0.233 | 0.210 | 0.237 | 0.176 | 0.132 | 0.101 | 0.102 | 0.079 | 0.084 | 0.075 | 0.076 | 0.079 | 0.072 |
| North America, High Income | Opioid | 0.957 | 0.025 | 0.340 | 0.174 | 0.408 | 1.684 | 3.499 | 4.342 | 4.193 | 3.653 | 2.884 | 1.843 | 0.998 | 0.435 | 0.182 | 0.105 | 0.062 | 0.046 | 0.023 |
| Asia, Central | Opioid | 0.163 | 0.085 | 0.443 | 0.469 | 0.502 | 0.456 | 0.353 | 0.509 | 0.339 | 0.236 | 0.227 | 0.142 | 0.143 | 0.076 | 0.069 | 0.043 | 0.033 | 0.088 | 0.025 |
| Europe, Central | Opioid | 0.116 | 0.137 | 0.470 | 0.410 | 0.386 | 0.608 | 1.026 | 0.831 | 0.502 | 0.245 | 0.166 | 0.098 | 0.071 | 0.053 | 0.032 | 0.024 | 0.017 | 0.012 | 0.007 |
| Europe, Eastern | Opioid | 0.426 | 0.226 | 1.053 | 1.061 | 1.050 | 2.226 | 2.308 | 2.497 | 1.020 | 0.607 | 0.409 | 0.320 | 0.267 | 0.160 | 0.114 | 0.067 | 0.048 | 0.040 | 0.022 |
| Sub-Saharan Africa, Central | Opioid | 0.017 | 0.011 | 0.030 | 0.017 | 0.023 | 0.037 | 0.033 | 0.026 | 0.021 | 0.019 | 0.016 | 0.015 | 0.016 | 0.011 | 0.007 | 0.005 | 0.004 | 0.004 | 0.004 |
| Sub-Saharan Africa, East | Opioid | 0.045 | 0.019 | 0.085 | 0.046 | 0.056 | 0.122 | 0.104 | 0.081 | 0.063 | 0.049 | 0.046 | 0.039 | 0.042 | 0.031 | 0.020 | 0.016 | 0.012 | 0.013 | 0.013 |
| Sub-Saharan Africa, Southern | Opioid | 0.222 | 0.412 | 0.233 | 0.339 | 0.351 | 0.694 | 0.320 | 0.170 | 0.129 | 0.158 | 0.185 | 0.183 | 0.194 | 0.173 | 0.129 | 0.095 | 0.079 | 0.059 | 0.055 |
| Sub-Saharan Africa, West | Opioid | 0.018 | 0.009 | 0.028 | 0.019 | 0.024 | 0.047 | 0.042 | 0.032 | 0.025 | 0.020 | 0.017 | 0.015 | 0.016 | 0.012 | 0.009 | 0.007 | 0.005 | 0.005 | 0.005 |
| North Africa / Middle East | Opioid | 0.122 | 0.057 | 0.214 | 0.108 | 0.154 | 0.247 | 0.263 | 0.281 | 0.369 | 0.181 | 0.230 | 0.166 | 0.170 | 0.146 | 0.097 | 0.082 | 0.073 | 0.051 | 0.061 |
| Asia, South | Opioid | 0.010 | 0.004 | 0.019 | 0.015 | 0.017 | 0.027 | 0.032 | 0.032 | 0.026 | 0.019 | 0.016 | 0.011 | 0.009 | 0.006 | 0.004 | 0.003 | 0.002 | 0.002 | 0.002 |
| Asia, East | Opioid | 0.061 | 0.038 | 0.390 | 0.143 | 0.212 | 0.142 | 0.240 | 0.162 | 0.118 | 0.076 | 0.078 | 0.058 | 0.043 | 0.039 | 0.020 | 0.015 | 0.025 | 0.008 | 0.008 |
| Asia, Southeast | Opioid | 0.012 | 0.003 | 0.034 | 0.025 | 0.022 | 0.026 | 0.033 | 0.028 | 0.027 | 0.022 | 0.017 | 0.013 | 0.011 | 0.008 | 0.008 | 0.007 | 0.004 | 0.004 | 0.003 |
| Oceania | Opioid | 0.069 | 0.031 | 0.140 | 0.102 | 0.109 | 0.180 | 0.232 | 0.147 | 0.140 | 0.095 | 0.072 | 0.068 | 0.057 | 0.038 | 0.030 | 0.026 | 0.026 | 0.029 | 0.055 |
| Caribbean | Opioid | 0.070 | 0.116 | 0.195 | 0.143 | 0.062 | 0.061 | 0.050 | 0.055 | 0.056 | 0.064 | 0.043 | 0.066 | 0.078 | 0.032 | 0.021 | 0.022 | 0.017 | 0.018 | 0.021 |
| Latin America, Andean | Opioid | 0.197 | 0.065 | 0.518 | 0.544 | 0.450 | 0.741 | 0.668 | 0.365 | 0.367 | 0.254 | 0.246 | 0.186 | 0.168 | 0.130 | 0.103 | 0.103 | 0.076 | 0.067 | 0.065 |
| Latin America, Central | Opioid | 0.074 | 0.046 | 0.224 | 0.127 | 0.162 | 0.173 | 0.212 | 0.170 | 0.148 | 0.124 | 0.097 | 0.072 | 0.055 | 0.047 | 0.032 | 0.033 | 0.032 | 0.028 | 0.031 |
| Latin America, Tropical | Opioid | 0.040 | 0.021 | 0.176 | 0.084 | 0.079 | 0.149 | 0.114 | 0.110 | 0.076 | 0.059 | 0.059 | 0.036 | 0.032 | 0.025 | 0.024 | 0.025 | 0.024 | 0.018 | 0.023 |
| Global | Cocaine | 0.001 | 0.000 | 0.001 | 0.001 | 0.001 | 0.002 | 0.002 | 0.002 | 0.002 | 0.002 | 0.002 | 0.002 | 0.001 | 0.001 | 0.001 | 0.000 | 0.000 | 0.000 | 0.000 |
| Asia Pacific, High Income | Cocaine | 0.001 | 0.001 | 0.001 | 0.001 | 0.004 | 0.003 | 0.006 | 0.006 | 0.006 | 0.004 | 0.002 | 0.001 | 0.001 | 0.001 | 0.000 | 0.000 | 0.000 | 0.000 | 0.000 |
| Australasia | Cocaine | 0.016 | 0.001 | 0.008 | 0.006 | 0.012 | 0.048 | 0.106 | 0.097 | 0.082 | 0.050 | 0.037 | 0.020 | 0.011 | 0.005 | 0.003 | 0.002 | 0.001 | 0.001 | 0.000 |
| Europe, Western | Cocaine | 0.003 | 0.001 | 0.002 | 0.001 | 0.004 | 0.013 | 0.024 | 0.025 | 0.017 | 0.008 | 0.005 | 0.002 | 0.001 | 0.001 | 0.000 | 0.000 | 0.000 | 0.000 | 0.000 |
| Latin America, Southern | Cocaine | 0.002 | 0.003 | 0.009 | 0.004 | 0.003 | 0.003 | 0.004 | 0.003 | 0.004 | 0.003 | 0.002 | 0.002 | 0.002 | 0.001 | 0.002 | 0.001 | 0.001 | 0.001 | 0.001 |
| North America, High Income | Cocaine | 0.014 | 0.000 | 0.006 | 0.003 | 0.006 | 0.022 | 0.050 | 0.066 | 0.071 | 0.055 | 0.043 | 0.023 | 0.014 | 0.006 | 0.003 | 0.002 | 0.001 | 0.001 | 0.000 |
| Asia, Central | Cocaine | 0.002 | 0.002 | 0.006 | 0.006 | 0.006 | 0.006 | 0.005 | 0.007 | 0.004 | 0.003 | 0.003 | 0.002 | 0.002 | 0.001 | 0.001 | 0.001 | 0.000 | 0.001 | 0.000 |
| Europe, Central | Cocaine | 0.001 | 0.001 | 0.004 | 0.004 | 0.004 | 0.005 | 0.009 | 0.008 | 0.005 | 0.002 | 0.001 | 0.001 | 0.001 | 0.000 | 0.000 | 0.000 | 0.000 | 0.000 | 0.000 |
| Europe, Eastern | Cocaine | 0.006 | 0.004 | 0.018 | 0.016 | 0.017 | 0.027 | 0.032 | 0.028 | 0.012 | 0.008 | 0.005 | 0.004 | 0.004 | 0.002 | 0.002 | 0.001 | 0.001 | 0.001 | 0.000 |
| Sub-Saharan Africa, Central | Cocaine | 0.000 | 0.000 | 0.000 | 0.000 | 0.000 | 0.000 | 0.000 | 0.000 | 0.000 | 0.000 | 0.000 | 0.000 | 0.000 | 0.000 | 0.000 | 0.000 | 0.000 | 0.000 | 0.000 |
| Sub-Saharan Africa, East | Cocaine | 0.000 | 0.000 | 0.000 | 0.000 | 0.000 | 0.000 | 0.000 | 0.000 | 0.000 | 0.000 | 0.000 | 0.000 | 0.000 | 0.000 | 0.000 | 0.000 | 0.000 | 0.000 | 0.000 |
| Sub-Saharan Africa, Southern | Cocaine | 0.002 | 0.003 | 0.002 | 0.003 | 0.005 | 0.005 | 0.003 | 0.002 | 0.001 | 0.002 | 0.003 | 0.002 | 0.002 | 0.002 | 0.002 | 0.001 | 0.000 | 0.001 | 0.000 |
| Sub-Saharan Africa, West | Cocaine | 0.000 | 0.000 | 0.000 | 0.000 | 0.000 | 0.000 | 0.000 | 0.000 | 0.000 | 0.000 | 0.000 | 0.000 | 0.000 | 0.000 | 0.000 | 0.000 | 0.000 | 0.000 | 0.000 |
| North Africa / Middle East | Cocaine | 0.002 | 0.001 | 0.004 | 0.002 | 0.003 | 0.004 | 0.005 | 0.005 | 0.007 | 0.003 | 0.004 | 0.002 | 0.003 | 0.002 | 0.001 | 0.001 | 0.001 | 0.001 | 0.001 |
| Asia, South | Cocaine | 0.000 | 0.000 | 0.000 | 0.000 | 0.000 | 0.000 | 0.000 | 0.000 | 0.000 | 0.000 | 0.000 | 0.000 | 0.000 | 0.000 | 0.000 | 0.000 | 0.000 | 0.000 | 0.000 |
| Asia, East | Cocaine | 0.001 | 0.001 | 0.008 | 0.003 | 0.004 | 0.003 | 0.006 | 0.003 | 0.002 | 0.001 | 0.002 | 0.001 | 0.001 | 0.001 | 0.000 | 0.000 | 0.000 | 0.000 | 0.000 |
| Asia, Southeast | Cocaine | 0.000 | 0.000 | 0.000 | 0.000 | 0.000 | 0.000 | 0.001 | 0.000 | 0.000 | 0.000 | 0.000 | 0.000 | 0.000 | 0.000 | 0.000 | 0.000 | 0.000 | 0.000 | 0.000 |
| Oceania | Cocaine | 0.001 | 0.001 | 0.002 | 0.001 | 0.001 | 0.002 | 0.003 | 0.002 | 0.002 | 0.001 | 0.001 | 0.001 | 0.001 | 0.001 | 0.000 | 0.000 | 0.000 | 0.000 | 0.001 |
| Caribbean | Cocaine | 0.001 | 0.001 | 0.002 | 0.002 | 0.001 | 0.000 | 0.001 | 0.001 | 0.001 | 0.001 | 0.000 | 0.001 | 0.001 | 0.000 | 0.000 | 0.000 | 0.000 | 0.000 | 0.000 |
| Latin America, Andean | Cocaine | 0.002 | 0.001 | 0.006 | 0.006 | 0.005 | 0.007 | 0.007 | 0.005 | 0.005 | 0.003 | 0.003 | 0.002 | 0.002 | 0.002 | 0.001 | 0.001 | 0.001 | 0.001 | 0.001 |
| Latin America, Central | Cocaine | 0.001 | 0.001 | 0.004 | 0.002 | 0.003 | 0.003 | 0.004 | 0.003 | 0.003 | 0.002 | 0.002 | 0.001 | 0.001 | 0.001 | 0.001 | 0.001 | 0.001 | 0.001 | 0.000 |
| Latin America, Tropical | Cocaine | 0.000 | 0.000 | 0.002 | 0.001 | 0.001 | 0.001 | 0.001 | 0.001 | 0.000 | 0.001 | 0.001 | 0.000 | 0.000 | 0.000 | 0.000 | 0.000 | 0.000 | 0.000 | 0.000 |
| Global | Amphetamine | 0.001 | 0.000 | 0.001 | 0.001 | 0.001 | 0.001 | 0.002 | 0.002 | 0.002 | 0.002 | 0.002 | 0.001 | 0.001 | 0.001 | 0.000 | 0.000 | 0.000 | 0.000 | 0.000 |
| Asia Pacific, High Income | Amphetamine | 0.001 | 0.001 | 0.001 | 0.001 | 0.003 | 0.003 | 0.006 | 0.005 | 0.005 | 0.003 | 0.002 | 0.001 | 0.001 | 0.001 | 0.000 | 0.000 | 0.000 | 0.000 | 0.000 |
| Australasia | Amphetamine | 0.018 | 0.002 | 0.009 | 0.007 | 0.013 | 0.053 | 0.118 | 0.108 | 0.091 | 0.056 | 0.041 | 0.022 | 0.012 | 0.006 | 0.003 | 0.002 | 0.001 | 0.001 | 0.000 |
| Europe, Western | Amphetamine | 0.003 | 0.001 | 0.002 | 0.002 | 0.005 | 0.017 | 0.031 | 0.031 | 0.022 | 0.011 | 0.006 | 0.003 | 0.001 | 0.001 | 0.001 | 0.000 | 0.000 | 0.000 | 0.000 |
| Latin America, Southern | Amphetamine | 0.002 | 0.003 | 0.008 | 0.004 | 0.003 | 0.003 | 0.004 | 0.003 | 0.004 | 0.003 | 0.002 | 0.001 | 0.001 | 0.001 | 0.001 | 0.001 | 0.001 | 0.001 | 0.001 |
| North America, High Income | Amphetamine | 0.009 | 0.000 | 0.005 | 0.002 | 0.004 | 0.013 | 0.031 | 0.042 | 0.048 | 0.035 | 0.027 | 0.013 | 0.008 | 0.004 | 0.002 | 0.001 | 0.001 | 0.000 | 0.000 |
| Asia, Central | Amphetamine | 0.002 | 0.001 | 0.005 | 0.005 | 0.005 | 0.004 | 0.004 | 0.005 | 0.003 | 0.002 | 0.002 | 0.001 | 0.001 | 0.001 | 0.001 | 0.000 | 0.000 | 0.001 | 0.000 |
| Europe, Central | Amphetamine | 0.001 | 0.001 | 0.004 | 0.004 | 0.004 | 0.005 | 0.009 | 0.008 | 0.005 | 0.002 | 0.001 | 0.001 | 0.001 | 0.000 | 0.000 | 0.000 | 0.000 | 0.000 | 0.000 |
| Europe, Eastern | Amphetamine | 0.004 | 0.003 | 0.013 | 0.011 | 0.012 | 0.017 | 0.022 | 0.017 | 0.008 | 0.005 | 0.004 | 0.003 | 0.003 | 0.002 | 0.002 | 0.001 | 0.001 | 0.000 | 0.000 |
| Sub-Saharan Africa, Central | Amphetamine | 0.000 | 0.000 | 0.000 | 0.000 | 0.000 | 0.000 | 0.000 | 0.000 | 0.000 | 0.000 | 0.000 | 0.000 | 0.000 | 0.000 | 0.000 | 0.000 | 0.000 | 0.000 | 0.000 |
| Sub-Saharan Africa, East | Amphetamine | 0.000 | 0.000 | 0.000 | 0.000 | 0.000 | 0.000 | 0.000 | 0.000 | 0.000 | 0.000 | 0.000 | 0.000 | 0.000 | 0.000 | 0.000 | 0.000 | 0.000 | 0.000 | 0.000 |
| Sub-Saharan Africa, Southern | Amphetamine | 0.002 | 0.002 | 0.001 | 0.003 | 0.005 | 0.004 | 0.003 | 0.001 | 0.001 | 0.001 | 0.003 | 0.001 | 0.002 | 0.002 | 0.002 | 0.001 | 0.000 | 0.001 | 0.000 |
| Sub-Saharan Africa, West | Amphetamine | 0.000 | 0.000 | 0.000 | 0.000 | 0.000 | 0.000 | 0.000 | 0.000 | 0.000 | 0.000 | 0.000 | 0.000 | 0.000 | 0.000 | 0.000 | 0.000 | 0.000 | 0.000 | 0.000 |
| North Africa / Middle East | Amphetamine | 0.002 | 0.001 | 0.005 | 0.002 | 0.004 | 0.005 | 0.006 | 0.006 | 0.008 | 0.004 | 0.005 | 0.003 | 0.003 | 0.003 | 0.002 | 0.001 | 0.001 | 0.001 | 0.001 |
| Asia, South | Amphetamine | 0.000 | 0.000 | 0.000 | 0.000 | 0.000 | 0.000 | 0.000 | 0.000 | 0.000 | 0.000 | 0.000 | 0.000 | 0.000 | 0.000 | 0.000 | 0.000 | 0.000 | 0.000 | 0.000 |
| Asia, East | Amphetamine | 0.001 | 0.001 | 0.008 | 0.003 | 0.004 | 0.003 | 0.006 | 0.003 | 0.002 | 0.001 | 0.002 | 0.001 | 0.001 | 0.001 | 0.000 | 0.000 | 0.000 | 0.000 | 0.000 |
| Asia, Southeast | Amphetamine | 0.000 | 0.000 | 0.000 | 0.000 | 0.000 | 0.000 | 0.001 | 0.000 | 0.000 | 0.000 | 0.000 | 0.000 | 0.000 | 0.000 | 0.000 | 0.000 | 0.000 | 0.000 | 0.000 |
| Oceania | Amphetamine | 0.001 | 0.001 | 0.002 | 0.001 | 0.001 | 0.002 | 0.003 | 0.002 | 0.002 | 0.001 | 0.001 | 0.001 | 0.001 | 0.001 | 0.000 | 0.000 | 0.000 | 0.000 | 0.001 |
| Caribbean | Amphetamine | 0.001 | 0.002 | 0.003 | 0.002 | 0.001 | 0.001 | 0.001 | 0.001 | 0.001 | 0.001 | 0.001 | 0.001 | 0.001 | 0.000 | 0.000 | 0.000 | 0.000 | 0.000 | 0.000 |
| Latin America, Andean | Amphetamine | 0.002 | 0.001 | 0.007 | 0.006 | 0.005 | 0.008 | 0.007 | 0.005 | 0.005 | 0.003 | 0.003 | 0.002 | 0.002 | 0.002 | 0.001 | 0.001 | 0.001 | 0.001 | 0.001 |
| Latin America, Central | Amphetamine | 0.001 | 0.001 | 0.003 | 0.001 | 0.001 | 0.002 | 0.002 | 0.002 | 0.002 | 0.001 | 0.001 | 0.001 | 0.001 | 0.001 | 0.000 | 0.000 | 0.000 | 0.000 | 0.000 |
| Latin America, Tropical | Amphetamine | 0.000 | 0.000 | 0.002 | 0.001 | 0.001 | 0.001 | 0.001 | 0.001 | 0.001 | 0.001 | 0.001 | 0.000 | 0.000 | 0.000 | 0.000 | 0.000 | 0.000 | 0.000 | 0.000 |
